# Low Temperature–ROS–Hormones Co-Regulation of Seed Dormancy Release and Germination in *Xanthoceras sorbifolium*

**DOI:** 10.3390/plants15182790

**Published:** 2026-09-11

**Authors:** Yifan Wang, Na An, Ying Chen, Qinxia Wu, Zhao Yang, Hao Cai, Jingjing Di

**Affiliations:** Co-Innovation Center for Sustainable Forestry in Southern China, College of Life Sciences, Nanjing Forestry University, Nanjing 210037, China

**Keywords:** seed dormancy release, *X. sorbifolium* seeds, low temperature storage, ROS, hormones, metabolome

## Abstract

Seed dormancy is an important adaptive mechanism in plants. However, some seeds, such as those of *Xanthoceras sorbifolium* (a valuable economic and medicinal species), exhibit strong dormancy, resulting in a very low natural germination rate. This work investigated the mechanism by which low temperatures (LT: −20 °C storage for 60 days) release seed dormancy and promote germination. This was achieved by examining seed germination conditions, applying scanning electron microscopy (SEM), and measuring physiological indicators of seeds and the hormone levels. Targeted metabolomic analysis of sugar and fatty acid metabolism was also performed. The results were as follows: (1) A high germination rate of 47.3% was observed under LT condition, compared to 32.7% at room temperature (RT: 25 °C). Of four germination methods, direct GMS (germination in moist sand) was the most effective. (2) The two stages of VI (after storage) and VII (on the 7th day of germination) were key stages to breaking the seed dormancy and triggering germination. At both stages, the high integrity of the seed shells and kernels—particularly, the kernels—was observed using SEM. The activities of SOD, CAT, and POD, as well as the levels of IAA and IPA, and the ratios of IAA/ABA and (IAA + GA3 + ZR + IPA)/ABA (tHor/ABA) were found to be higher, especially in stage VII, where tHor/ABA increased by 62.7%, while ABA decreased by 23.2% in the LT treatment compared to the RT treatment. An optimal germination condition (GMS) created a suitable microenvironment, and this could have maintained highly active antioxidant enzymes and kept H_2_O_2_ (one of reactive oxygen species, or ROS) within signal transduction levels, and cross-talk to hormones. These changes (enzymes, hormones, ROS, and microenvironment) ensured the seeds reaching an optimal state for dormancy release in the VI stage, and facilitated the seed germination in the VII stage. (3) LT treatment promoted the degradation of starch and fats in the seeds. Significant accumulations of eight soluble sugars, such as glucose, D-fructose, and trehalose, as well as three fatty acids, such as Cis-11,14,17-eicosatrienoic acid (C20-3n3) and γ-linolenic acid (C18-3n6), were observed, while two soluble sugars and one fatty acid decreased under LT conditions. In conclusion, low temperature, as an external signal, together with ROS and GA-ABA-IAA etc. the internal signals co-regulated the seed dormancy release and germination. The moist-sand microenvironment was also a key factor in awakening the embryos of *X. sorbifolium* seeds.

## 1. Introduction

*Xanthoceras sorbifolium* Bunge is a deciduous and woody oil-bearing tree, belonging to the Sapindaceae family. Its seeds contain a high content (55–65%) of oil, with 93% unsaturated fatty acids (UFAs), and is considered a high-quality cooking oil [1,2]. The seed UFA contains about 40% oleic acid, 30% linoleic acid, and 5% nervonic acid [3,4]. As an extremely rare component in plant oils, nervonic acid could enhance people’s immunity, support infant brain development, and have the effects of treating cancer and tumors, as well as alleviating Alzheimer’s disease. Therefore, the oil of *X. sorbifolium* seeds could be used in medical health products, edible oils, and cosmetics, and also as a raw material for biofuel oil, in addition to possessing multiple industrial values [3,5,6,7]. *X. sorbifolium* trees are mainly distributed in Northwestern China, in regions such as Shaanxi, Shanxi, Hebei, Gansu, Inner Mongolia, Shandong, etc. [7,8]. These trees are also characterized by their tolerance of cold, drought, poor soil, and saline–alkali conditions, and have gained global recognition as valuable economic and medicinal species [6,8,9].

The quality of forest tree seeds is one of the key factors determining breeding efficiency, variety improvement, and the conservation of genetic resources [10]. However, the seeds of *X. sorbifolium* are dormant and have a natural germination rate that is only 6–20% [2,11]. This presents challenges for the cultivation of *X. sorbifolium* seedlings, and the development and promotion of new varieties. Therefore, it is crucial to study the mechanism of dormancy in *X. sorbifolium* seeds and measures to break dormancy.

Seed dormancy is a complex physiological and biochemical process that prevents seeds from germinating even when conditions are favorable. Therefore, seeds can only germinate when dormancy is released [12,13]. The process of releasing seed dormancy involves the degradation of starch, lipids, and proteins into soluble sugars, fatty acids, and amino acids to enable seed germination [14,15]. Recent research indicates that seed dormancy is primarily caused by five factors: (1) environmental factors (temperature, moisture, oxygen, light, etc.); (2) seed coat barriers (thick seed coat, presence of inhibitory substances, etc.), (3) physiological issues after-ripening (accumulation of large amounts of storage substances such as starch and fats); (4) the presence of germination-inhibiting substances (such as abscisic acid); and (5) incomplete embryo development, as well as composite dormancy (a combination of the above factors) [16,17,18,19]. For example, the thick, hard seed coats of *Phoebe sheareri* contained germination inhibitors, which were the main factor inhibited seed germination [20]; and Ginkgo seeds either lacked an embryo at harvest or had an embryo that was too small and underdeveloped to germinate [21]. The dormancy of *Phellodendron* seeds was primarily caused by mechanical constraints and barriers to water and air permeability in the pericarp and seed coat, as well as the presence of endogenous inhibitory substances, such as highly active vanillic acid in the pericarp [22,23].

Hormones such as gibberellin (GA), auxin (AUX), cytokinin (CTK), abscisic acid (ABA), ethylene (ET), and jasmonate (JA) are the key internal factors regulating seed dormancy and germination [24,25,26]. Among them, ABA plays a dominant role in maintaining seed dormancy, while GA promotes the release of seed dormancy by antagonizing ABA-mediated signaling and activating physiological processes [27].

Reactive oxygen species (ROS) such as superoxide anions (O_2_^•−^), hydrogen peroxide (H_2_O_2_), and hydroxyl radicals (•OH) are now recognized as necessary regulators of seed dormancy. However, excessive ROS such as hydrogen peroxide (H_2_O_2_) also could cause oxidative damage and inhibit seed germination [28,29]. The optimal level of ROS is considered the ‘oxidative window’ for germination, as both excessive and insufficient ROS could inhibit seed germination [27,30]. Therefore, maintaining homogeneous ROS and reducing oxidative stress are important measures for breaking seed dormancy during seed storage [31]. Antioxidant enzymes such as superoxide dismutase (SOD), peroxidase (POD), and catalase (CAT), as well as antioxidant substances such as ascorbic acid (AsA), glutathione (GSH), etc., could eliminate ROS and maintain cellular oxidation balance [19,32]. Hence, ROS, antioxidant enzymes, and hormones are key regulatory molecules in the process of seed dormancy release. However, the mechanism of cross-talk among ROS, antioxidant systems, and hormones during the seed dormancy release process remains unclear.

Temperature is one of the most important environmental factors affecting seed germination and could influence seed dormancy and longevity [33]. Cold storage could break seed dormancy and increase the germination rate. For instance, a high germination rate was observed in rice seeds stored at −20 °C for 30 years, indicating that low temperature could improve retention of viability [34]. There are several reports on the germination of *X. sorbifolium* seeds under cold storage conditions. The seed germination rate of *X. sorbifolium* significantly increased after they were stored in snow or sand at low temperatures for 90 days [35]. Storing the seeds at −20 °C for 10 months or ‘dingling sowing’ (sowing when the ground is half frozen and half thawed in early spring) also significantly improved the germination rate of *X. sorbifolium* seeds [36,37]. Similarly, the germination rate of *X. sorbifolium* seeds was significantly improved by storage at −20 °C for 15 days [38]. Our recent research has also shown that storing seeds at a low temperature (LT: −20 °C) could significantly improve the seed germination rate [2]. Researches have indicated that low temperatures could break seed dormancy by degrading substances that inhibit germination during cold stratification or cold storage, thereby completed physiological after-ripening and promoting germination [39]. However, the physiological and molecular mechanisms by which low temperature breaks seed dormancy in *X. sorbifolium* remain unclear.

In this work, we investigated the mechanism by which LT triggers the release of dormancy and germination in *X. sorbifolium* seeds. Our research involved studying the seeds’ water absorption properties, electrical conductivity, and ultrastructural scanning, as well as measuring oxidative stress and analyzing the targeted metabolomics and hormones of the seeds. These findings will provide theoretical and technical support for breeding superior *X. sorbifolium* varieties and researching seed dormancy mechanisms.

## 2. Results

### 2.1. Effect of LT Storage on Seed Germination

**Figure 1:**
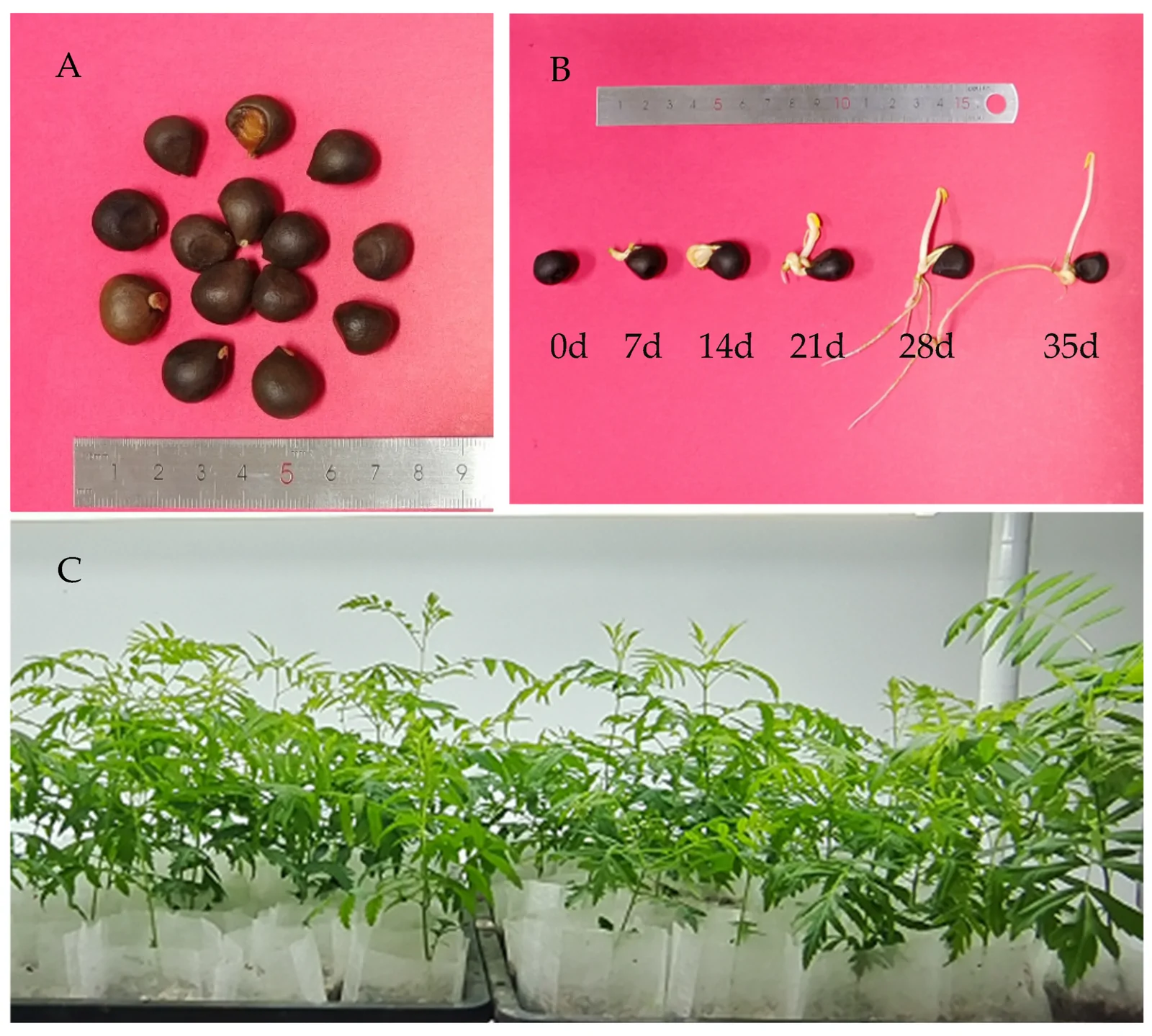
*X. sorbifolium* seeds and their germination phenotypes and seedlings. (**A**) the seeds before storage, (**B**) germination period seeds after storage, and (**C**) seedlings after transplanting 30 days.

**Figure 2:**
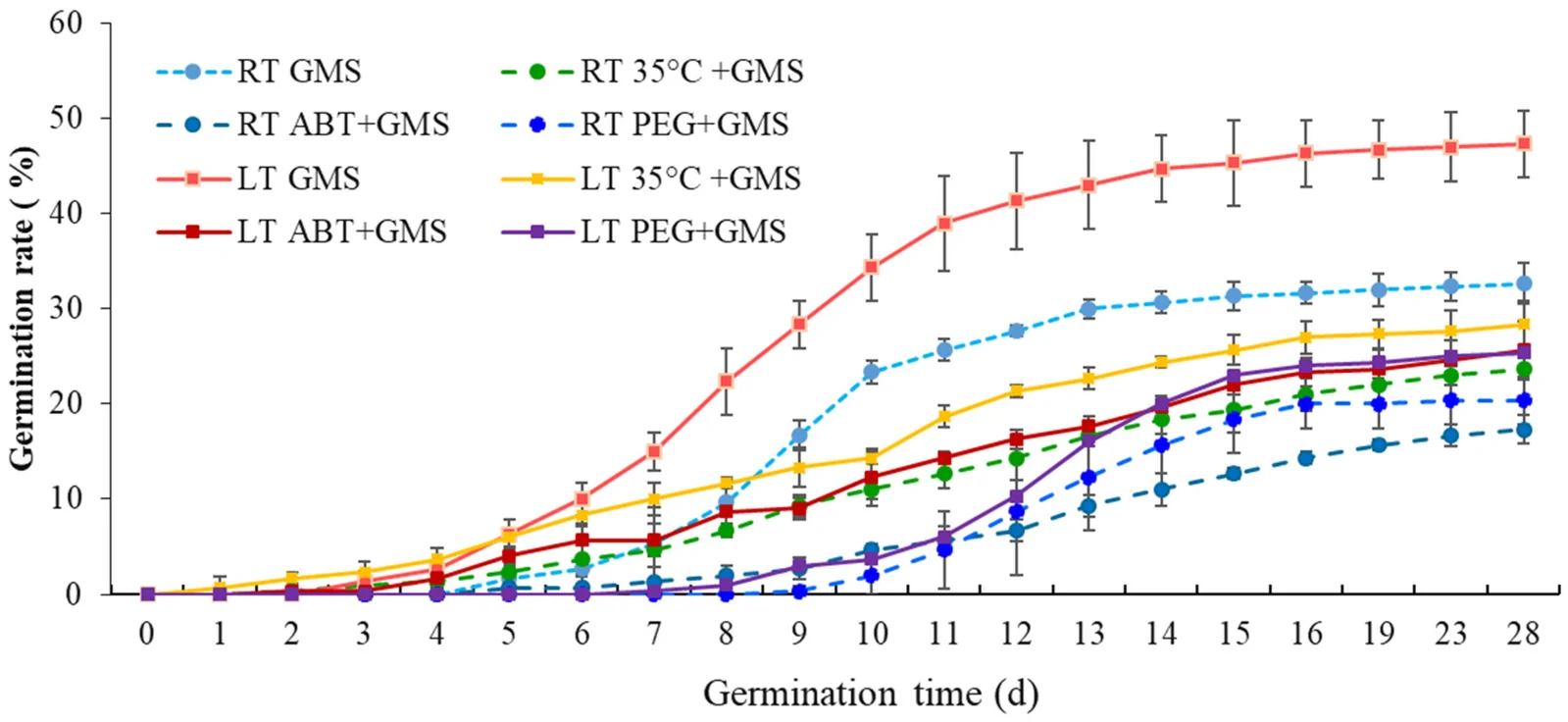
Cumulative germination frequency of *X. sorbifolium* seeds subjected to different germination treatments under two storage conditions. GMS, direct germination in moist sand after storage; 35 °C + GMS, seeds soaked in 35 °C water for 5 days after storage, then germinated in moist sand (the same below); ABT + GMS, seeds soaked in 200 mg/L ABT for 5 days after storage; PEG + GMS, seeds soaked in 5% PEG for 5 days after storage; RT, seeds stored at room temperature for 60 days; LT, seeds stored at −20 °C for 60 days.

**Figure 3:**
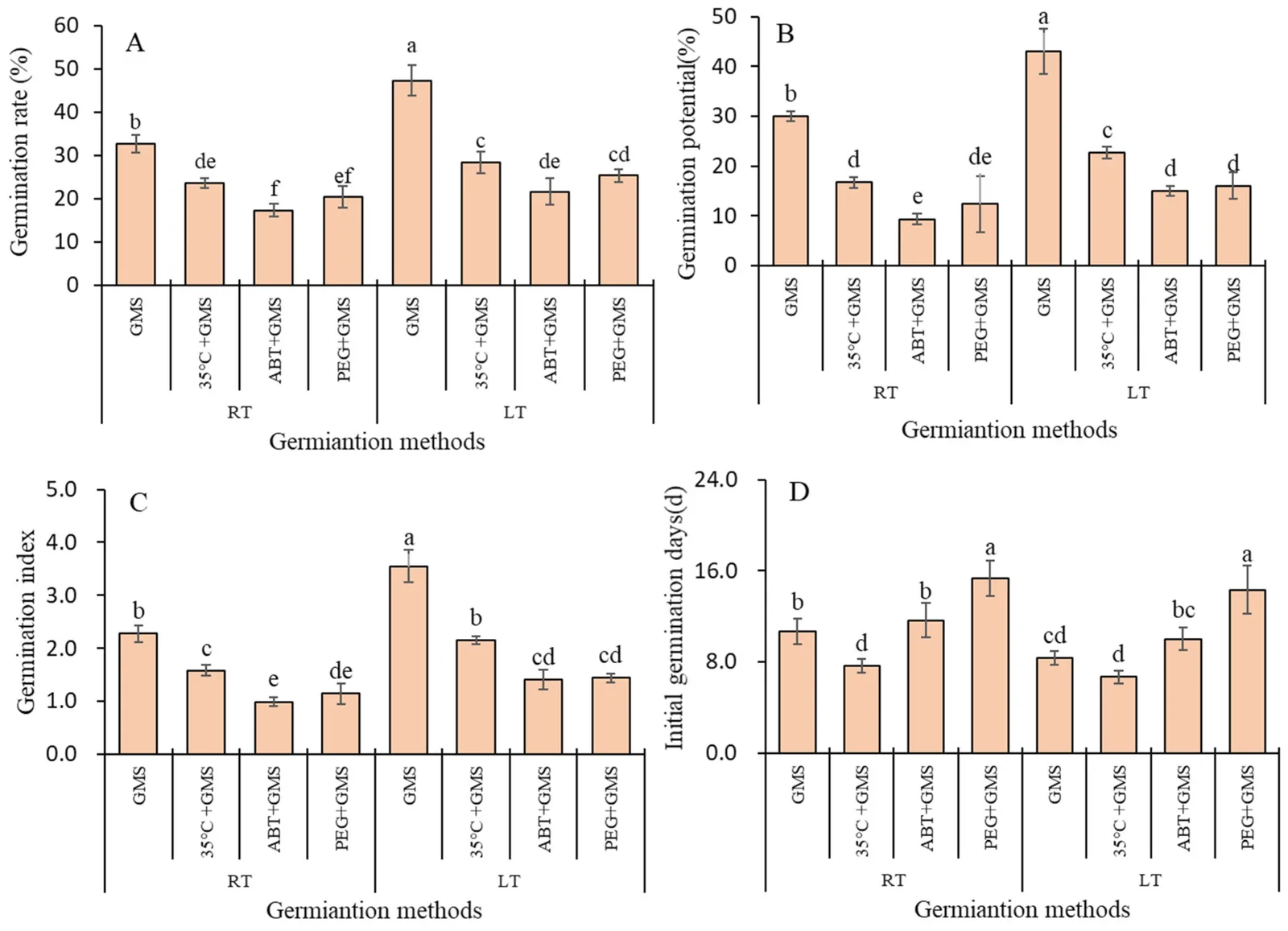
Different treatments on seed germination parameters of *X. sorbifolium*. Data represent the mean ± SD of three biological replicates. Different lowercase letters indicate significant differences between germination treatments and two storages (*p* < 0.05). (**A**): Germination rate; (**B**): Germination potential; (**C**): Germination index; (**D**): Initial germination days. GMS, direct germination in moist sand after storage; 35 °C + GMS, seeds soaked in 35 °C water for 5 days after storage, then germinated in moist sand (the same below); ABT + GMS, seeds soaked in 200 mg/L ABT for 5 days after storage; PEG + GMS, seeds soaked in 5% PEG for 5 days after storage; RT, seeds stored at room temperature for 60 days; LT, seeds stored at −20 °C for 60 days.

Figure 1 shows the *X. sorbifolium* seeds before storage (Figure 1A), during germination (Figure 1B), and as seedlings one month after transplanting (Figure 1C). After storing the seeds at −20 °C (LT) and room temperature at 25 °C (RT) for 60 days, four different germination treatments were applied to them, and the results are shown in Figure 2 and Figure 3. Among the four treatments, the cumulative germination frequency, germination rate, germination potential, and germination index under direct germination in moist-sand treatment (GMS) was the highest when stored in LT or RT conditions, while the effect of the ABT treatment was the lowest. Furthermore, the GMS and 35 °C + GMS treatments produced the earliest germination, with seeds germinating from the first or second day. In contrast, the ABT + GMS and 5% PEG + GMS treatments produced germination from the seventh day (Figure 2 and Figure 3). The survival rate, height, main root length, and dry and fresh weights of the seedlings treated with 5% PEG + GMS were slightly better than those treated with GMS treatment alone. However, there was no significant difference between the two treatments (*p* < 0.05). The above parameters for the 35 °C + GMS and ABT + GMS treatments were less effective than the first two treatments. Additionally, all the indicators were significantly higher for LT storage than for RT storage. For example, the germination rate of LT condition (47.3%) was higher than that of the RT condition (32.7%) (Figure 2 and Figure 3). Therefore, freezing storage at −20 °C combined with direct moist-sand germination (GMS) was an effective method for germinating in *X. sorbifolium* seeds (Table 1).

### 2.2. Effect of LT Storage on Seed Ultrastructure

**Figure 4:**
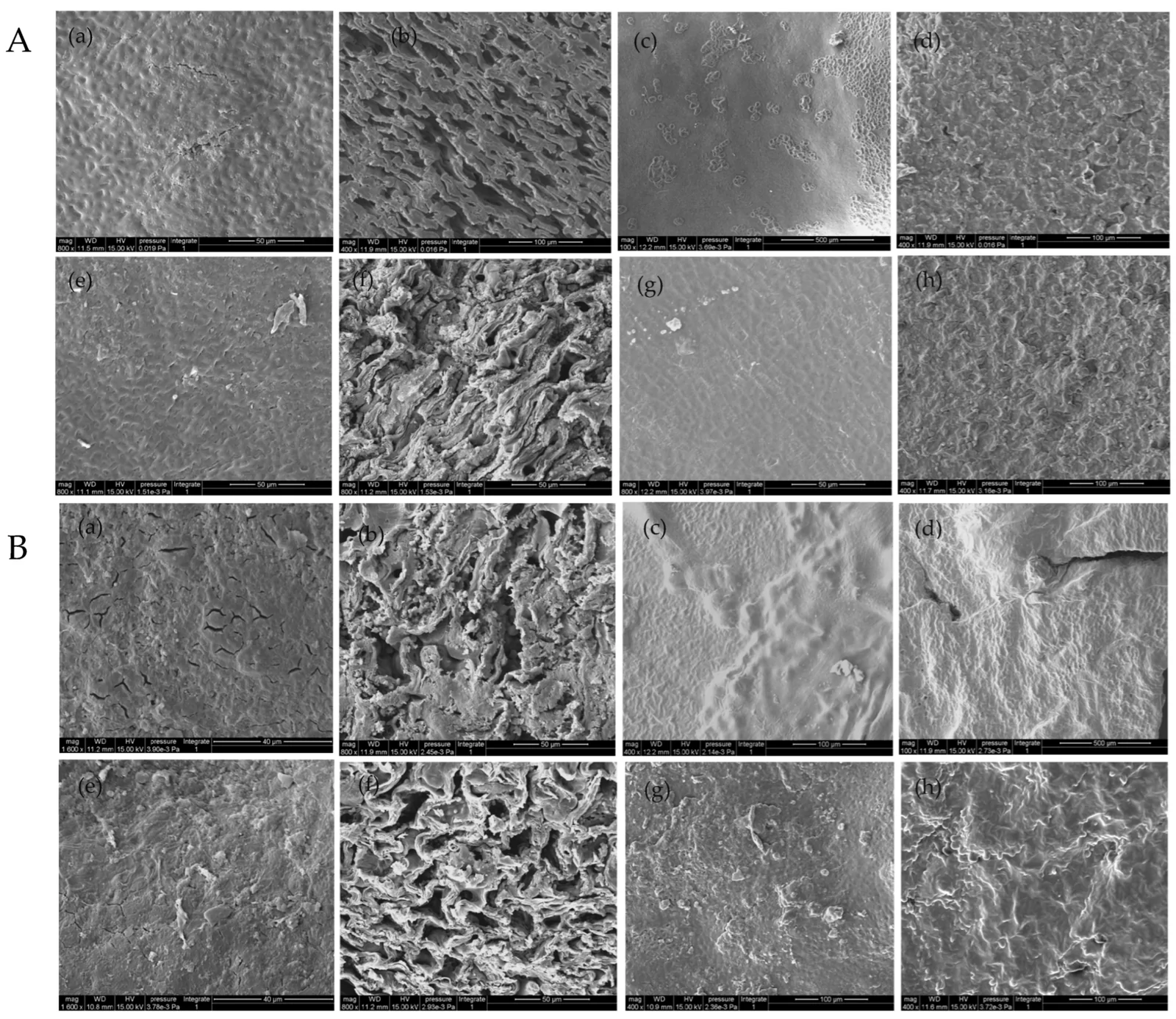
SEM observation under two storage methods. (**A**) Ultrastructure after 60 days of storage (stage VI): (**a**–**d**) storage at room temperature (RT) and (**e**–**h**) storage at low temperature (LT). (**B**) Ultrastructure of seeds germination for 7 days after 60 days of storage (stage VII): (**a**–**d**) seeds stored at room temperature (RT) and (**e**–**h**) seeds of stored at low temperature (LT). (**a**,**e**) Seed shell; (**b**,**f**) side view of seed shell; (**c**,**g**) outer surface of seed kernels; and (**d**,**h**) inner surface of seed kernels.

After 60 days of storage at low temperatures, the ultrastructure of the seeds was examined using a scanning electron microscope (SEM), and the results are shown in Figure 4. In LT storage, the seed shells were plump, smooth, and tight, with few gaps, and the surface of the seed kernel was smooth, with an intact internal structure (Figure 4A(e–h)). This indicated that low-temperature storage could effectively preserve the seed shells and kernels of *X. sorbifolium* and reduce moisture loss, and minimize damage to the seed. In contrast, seeds stored at RT storage for 60 days exhibited noticeable wrinkling on the surface of the shell surface, which was covered with fine cracks, and gaps appeared on the sides. Both the outer and inner surfaces of the seed kernel were rough, with distinct cracks, indicating that the room temperature storage caused damage to the shells and kernels of seeds (Figure 4A(a–d)).

On the 7th day of germination, the shells of *X. sorbifolium* seeds were slightly rough on the surface but showed no cracks. The gaps on the sides of the shells were uniform. The seed kernels also exhibited symptoms such as roughness, protrusions, and depressions on the surface and interior under LT storage. However, they remained relatively intact and smooth compared to seeds stored at LT (Figure 4B(e–h)). In contrast, on the 7th day of germination at RT condition, cracks had appeared on the seed kernel, with large gaps and fissures on the sides stored. The surface and interior of the seed kernel exhibited distinct ridge-like striations under room storage (RT), indicating significant damage (Figure 4B(a–d)).

### 2.3. Effect of LT Storage on Seeds’ Water Absorption

**Figure 5:**
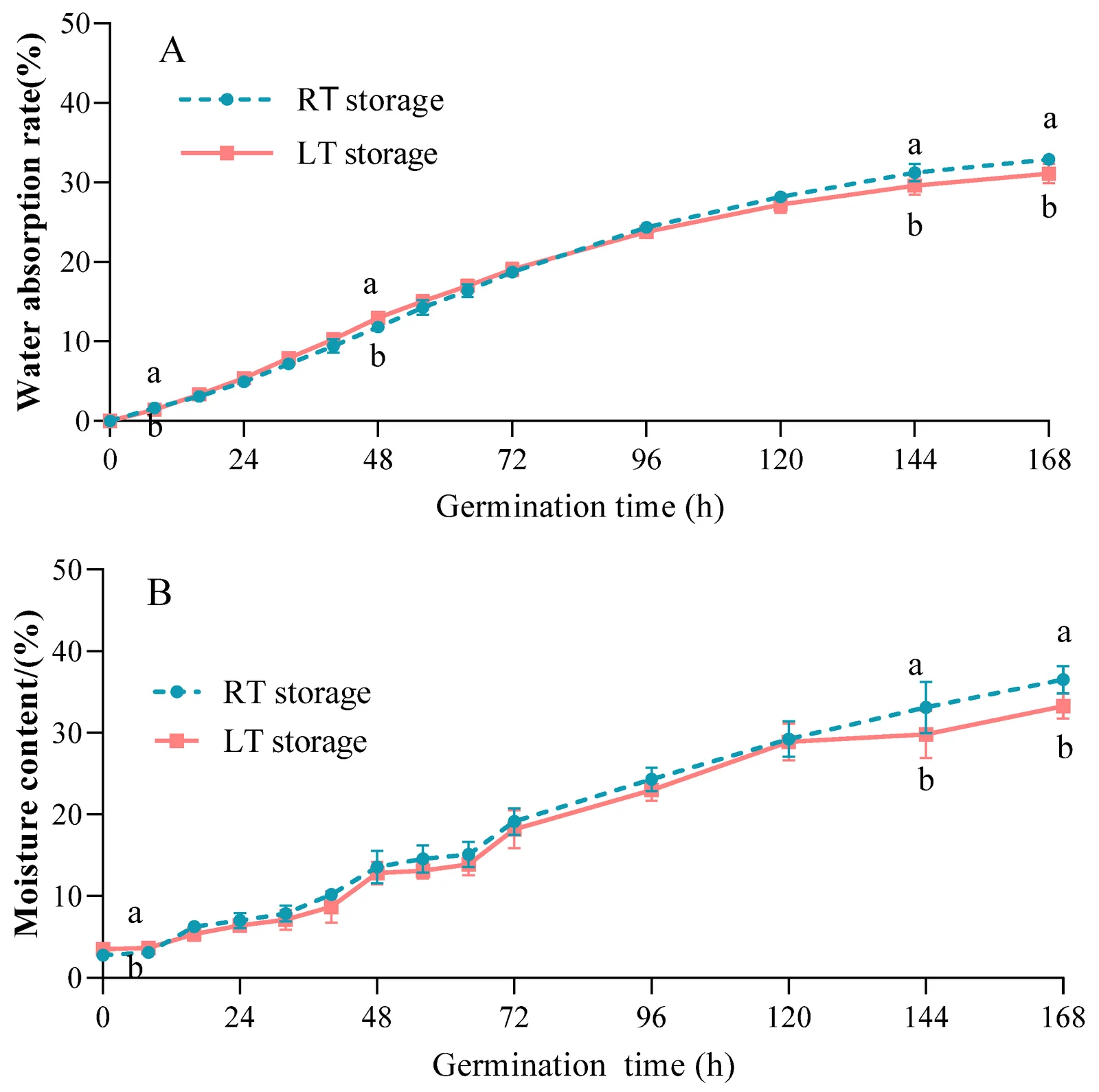
Effects of two storage methods on water absorption rate (**A**) and moisture content (**B**) of *X. sorbifolium* seed. Data represent the mean ± SD of three biological replicates. Lowercase letters (a, b) indicate significant differences between two storage methods at the same germination time (*p* < 0.05). The absence of statistical lettering at certain germination time point indicates that there is no significant difference between RT and LT in these time points (they should be labeled as a, a).

After storing the *X. sorbifolium* seeds for 60 days, they were germinated in moist sand. Differences in the water absorption rate (WAR) and moisture content (MC) of the seeds were observed (Figure 5). At the end of storage (stage VI), MC at stored LT condition exhibited significantly higher than that of seeds stored at RT condition. During the 168 h (stage VII) seed germination processes, the WAR (Figure 5A) and MC (Figure 5B) of the seeds showed an upward trend, appearing as an ‘S-shaped’ curve. Water uptake and moisture content were both relatively slow within the first 0–72 h, with increasing gradually between 96 and 168 h. For example, after 72 h, the WAR or MC values for seeds stored at RT and LT were 18.74% and 19.05%, or 19.12% and 18.19%, respectively, and no significant difference was observed between RT and LT conditions (*p* > 0.05). However, differences emerged from 120 h, with significant differences observed between 144 and 168 h (6th–7th day of germination). Compared to RT storage, the WAR and MC of LT samples were decreased by 9.92% and 8.95%, respectively, after 168 h of germination (Figure 5).

### 2.4. Effects of LT Storage on the REC of the Shells and Kernels of Seed

**Figure 6:**
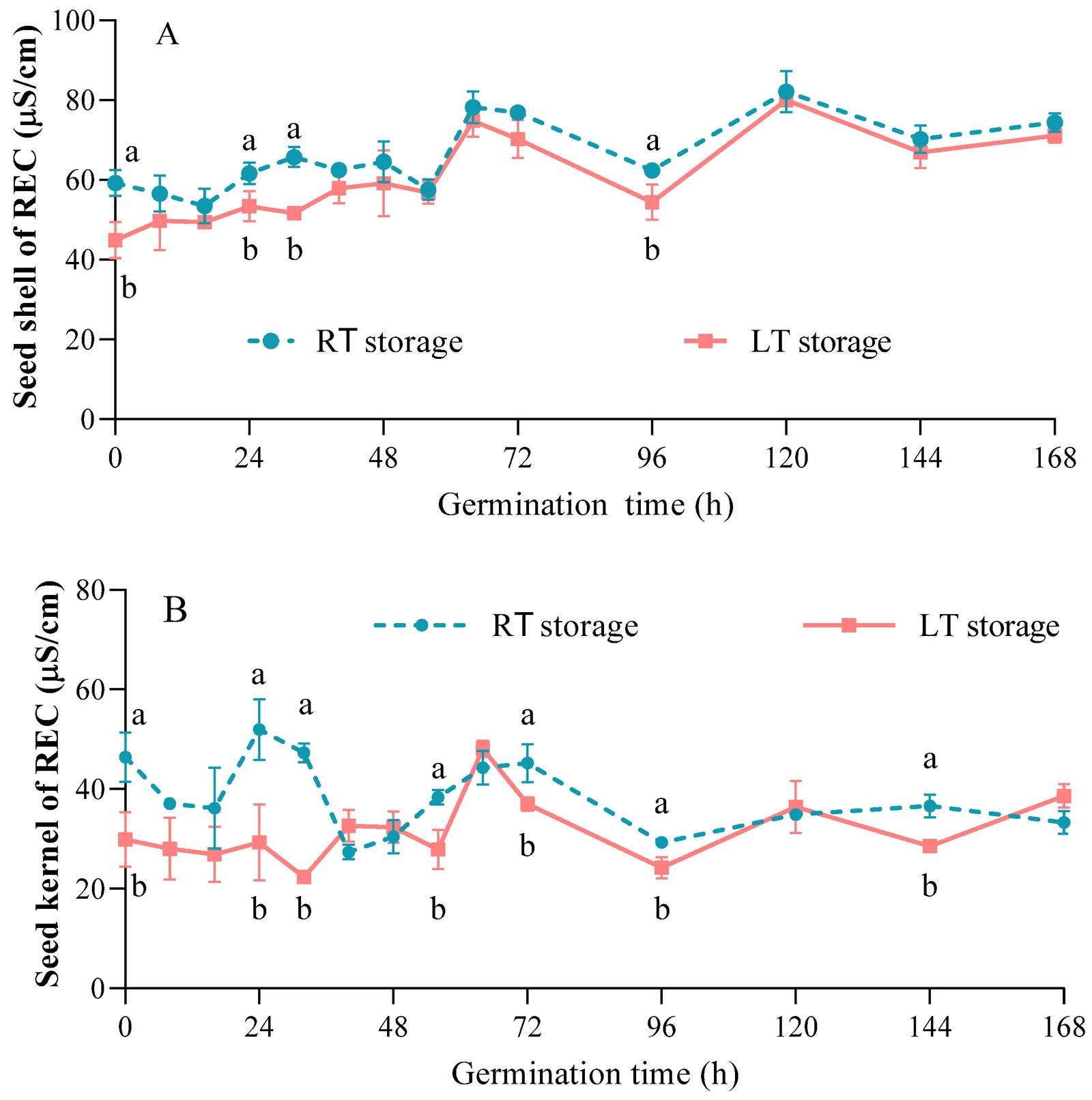
Effects of two storage methods on REC of seed shells (**A**) and kernels (**B**) of *X. sorbifolium* seed. Data represent the mean ± SD of three biological replicates. Lowercase letters (a, b) indicate significant differences between two storage methods at the same germination time (*p* < 0.05). The absence of statistical lettering at certain germination time point indicates that there is no significant difference between RT and LT in these time points (they should be labeled as a, a).

Following LT storage, the relative electrical conductivity (REC) of both the seed shells and kernels was significantly lower than that of seed stored at RT. During germination, significant differences in REC were also observed between the two storage conditions (Figure 6A). Fluctuating changes in REC were observed between 0 and 168 h, with two peaks at 72 and 120 h, respectively. Overall, the REC of seeds stored in LT condition was lower than that of seeds stored at the RT condition. Significant differences were primarily observed in the shells between 0 and 72 h, especially at 0 h (stage VI: after 60 days of storage), 8, 24, 32, and 96 h. For example, the REC values of LT samples were 21.5% (at 32 h) and 12.8% (at 96 h) lower than that of RT samples (Figure 6A).

The REC trend in seed kernels was similar to that in the seed shell: the REC was lower in the LT group than in the RT group throughout the 7-day germination period, with sharp fluctuations occurring between 0 (stage VI) and 48 h. Significant differences were observed at 0–32 h, 96 h, and 144 h in kernels, with the greatest differences observed at 0 h and 24 h. The LT treatment showed reductions of 35.6% and 43.6%, respectively, compared to the RT treatment. By 168 h, no significant difference between the two groups was found (Figure 6B).

### 2.5. Effects of LT Storage on the Antioxidant Indexes of the Seed

**Figure 7:**
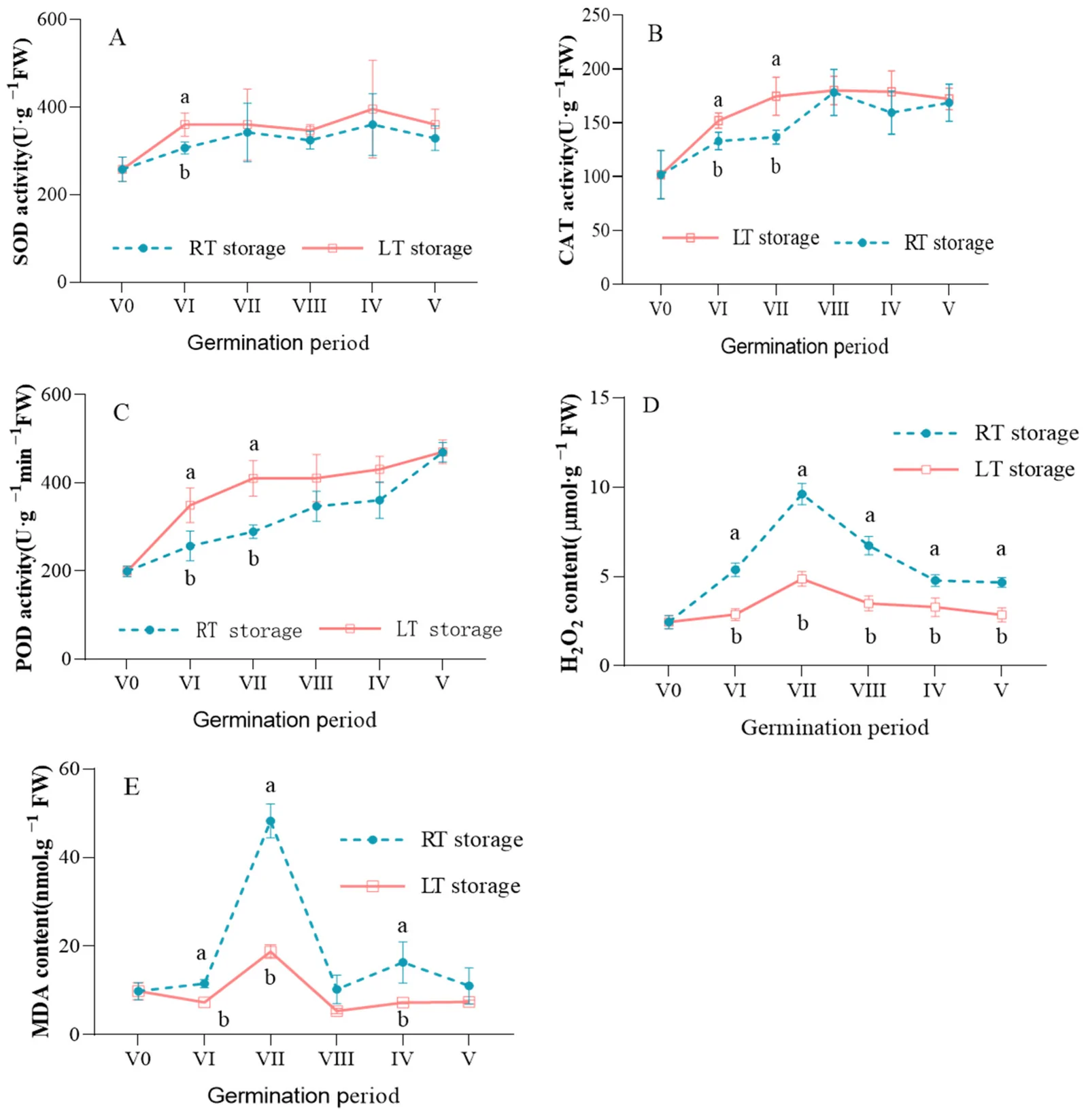
Effects of low-temperature storage on the activities of SOD (**A**), POD (**B**), CAT (**C**), H_2_O_2_ (**D**), and MDA (**E**) content during the storage and germination process of *X. sorbifolium* seeds. Data represent the mean ± SD of three biological replicates. Lowercase letters (a, b) indicate significant differences between two storage methods at the same germination time (*p* < 0.05). The absence of statistical lettering at certain germination time point indicates that there is no significant difference between RT and LT in these time points (they should be labeled as a, a).

During the storage and germination of *X. sorbifolium* seeds, the activities of the antioxidant enzymes (SOD, CAT, and POD) were observed to change significantly during germination between LT and RT treatments (Figure 7). The SOD activity in seeds treated at the LT condition remained higher than in the RT condition throughout the germination period. However, a significant difference was observed only on day 0 (stage VI), with SOD activity in the LT-treated seeds being 17.4% higher than that in RT-treated seeds. Changes in POD activity were greater than those in SOD. During the 0–21-day germination, POD activity was significantly higher in the LT samples than in the RT samples, with the greatest difference observed at stage VII (7th day of germination), as the LT-treated samples showed a 41.9% increase compared to the RT-treated samples. By stage V (28th day), there was no longer any difference in POD activity between the two groups. Changes in CAT enzyme activity were similar to those observed for SOD. Although enzyme activity was higher in the LT treatment group than in the RT treatment group during germination, a significant difference was only observed in stages VI and VII, with an increase of 14.2% and 27.8% compared to the RT treatment, respectively (Figure 7A–C).

The two storage methods also resulted in variations in H_2_O_2_ content. From VI to V (after storage and 28 days after germination), the H_2_O_2_ content in the room-temperature-treated (RT) seeds was significantly higher than that in the low-temperature-treated (LT) seeds. At time points VI–VII in particular, the H_2_O_2_ content was higher 87.0% and 97.4% than in the LT treatment (*p* < 0.05, Figure 7D). This indicates that storing the seeds at room temperature led to a significant increase in reactive oxygen species in the seeds.

Malondialdehyde (MDA) is one of the major products of lipid peroxidation in plant membranes; an increase in MDA content usually suggests deteriorating membrane damage. As shown in Figure 7E, MDA levels in *X. sorbifolium* seeds stored at RT condition were higher than those in LT condition at stages V1–V (five stages). This difference was particularly pronounced at stage VII of seed germination, when MDA levels peaked in the RT group, reaching 148.1% higher levels than in the LT condition at the same stage. This suggests that membrane damage was most severe on the 7th day of seed germination (stage VII), and the storing *X. sorbifolium* seeds in LT condition could reduce membrane damage.

### 2.6. Changes in Hormone Levels During LT Storage and Germination

**Figure 8:**
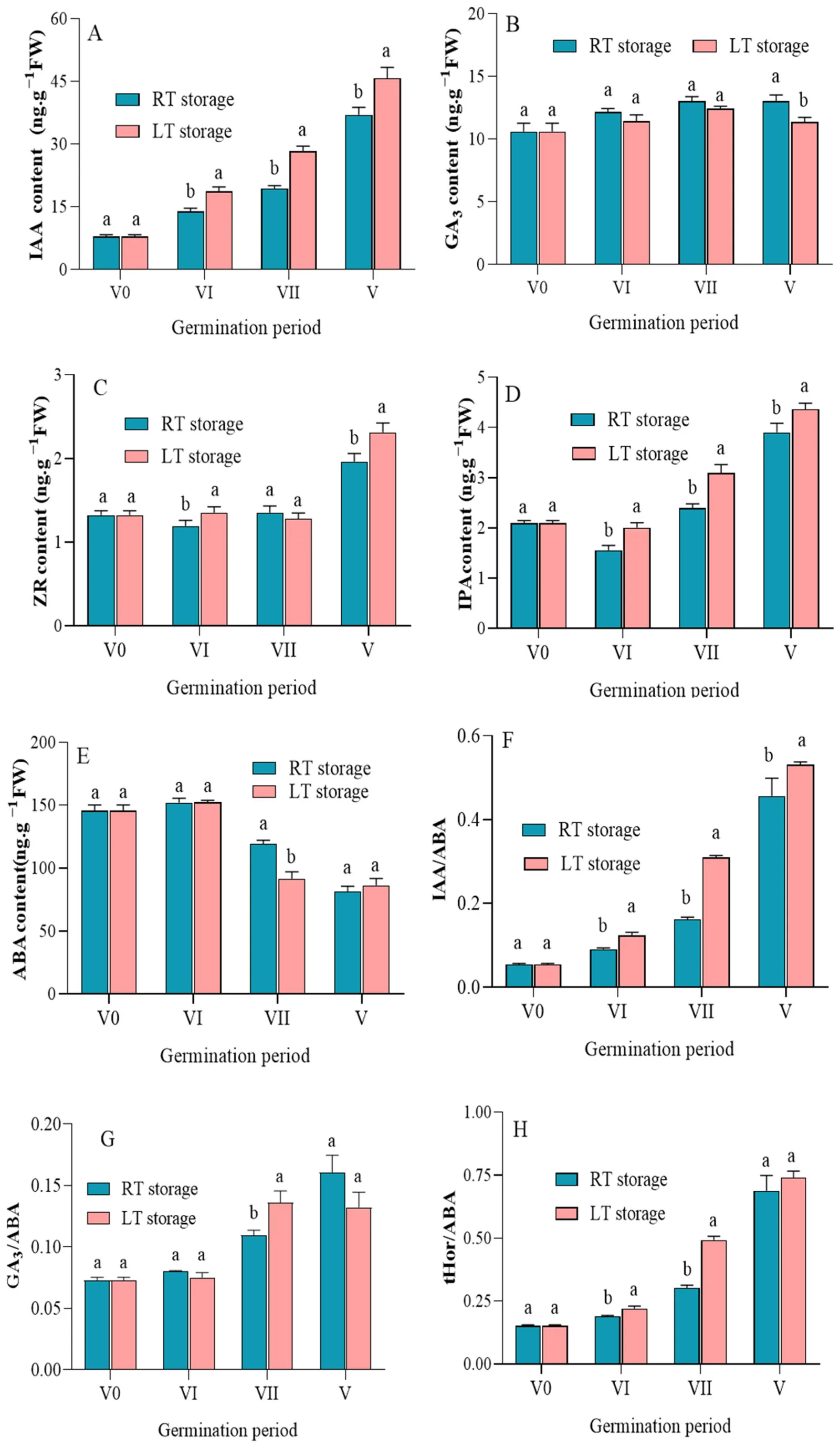
Effects of two storage methods on endogenous hormone contents during the storage and germination process of *X. sorbifolium* seeds. (**A**) IAA; (**B**) GA3; (**C**) ZR; (**D**) IPA; (**E**) ABA; (**F**) IAA/ABA; (**G**) GA3/ABA; and (**H**) tHor/ABA (IAA + GA3 + ZR + IPA: tHor). Data represent the mean ± SD of three biological replicates. Different lowercase letters indicate significant differences between two storage methods at the same germination period (*p* < 0.05).

The two storage methods also affected the hormone levels in the seeds (Figure 8). Throughout the germination period, the levels of IAA, IPA, GA, IAA/ABA, and tHor/ABA (tHor = IAA + GA3 + ZR + IPA) increased steadily, while ABA showed a downward trend. Compared with seeds stored at RT, the levels of IAA and IPA in *X. sorbifolium* seeds increased significantly after being LT-treated and during seed germination. After 60 days of storage (stage VI), on the 7th day of germination (stage VII) and on the 28th day after germination (stage V), the hormone content of seeds stored at LT was higher than that of seeds stored at RT during the three periods: 35.1%, 46.2%, and 23.7% for IAA; 28.9%, 29.2%, and 11.8% for IPA; and at 34.9%, 90.5%, and 16.4% for IAA/ABA, respectively. The values of ZR increased by 13.3% and 17.6% in stages VI and V under LT treatment compared to the RT treatment, respectively (Figure 8A,C,D,F).

Unlike IAA, the levels of GA3 and ABA exhibited different patterns. GA3 levels in the LT treatment decreased slightly by 6.0% and 12.9% at stages VI and V compared to the RT treatment, respectively. In contrast, ABA levels remained unchanged after RT storage (VI); however, at stage VII, the ABA level decreased significantly by 23.2% compared to the RT treatment. The GA3/ABA ratio showed no significantly difference in the LT treatment at stage VI, an increase of 24.5% at stage VII, and a decrease of 17.7% at stage V compared to the RT treatment (Figure 8B,E,G). However, the tHor/ABA ratio was significantly higher in the seeds stored at LT than that at RT during stages VI, VII, and V, with respective increases of 16.3%, 62.7%, and 7.4% (Figure 8H).

### 2.7. Metabolomic Analysis of Sugars and Fatty Acids During Seed Germination

A targeted metabolomics analysis of fatty acids and sugars in seeds was conducted after 7 days of germination following 60 days of storage under two different conditions (RT and LT). Principal component analysis (PCA) of metabolites explained 34.55% and 46.34% of the variance, respectively. Significant metabolic differences were observed between the LT and RT storage groups, while the three replicate samples clustered closely together, indicating the reproducibility and reliability of the data (Figure S1). 

**Figure 9:**
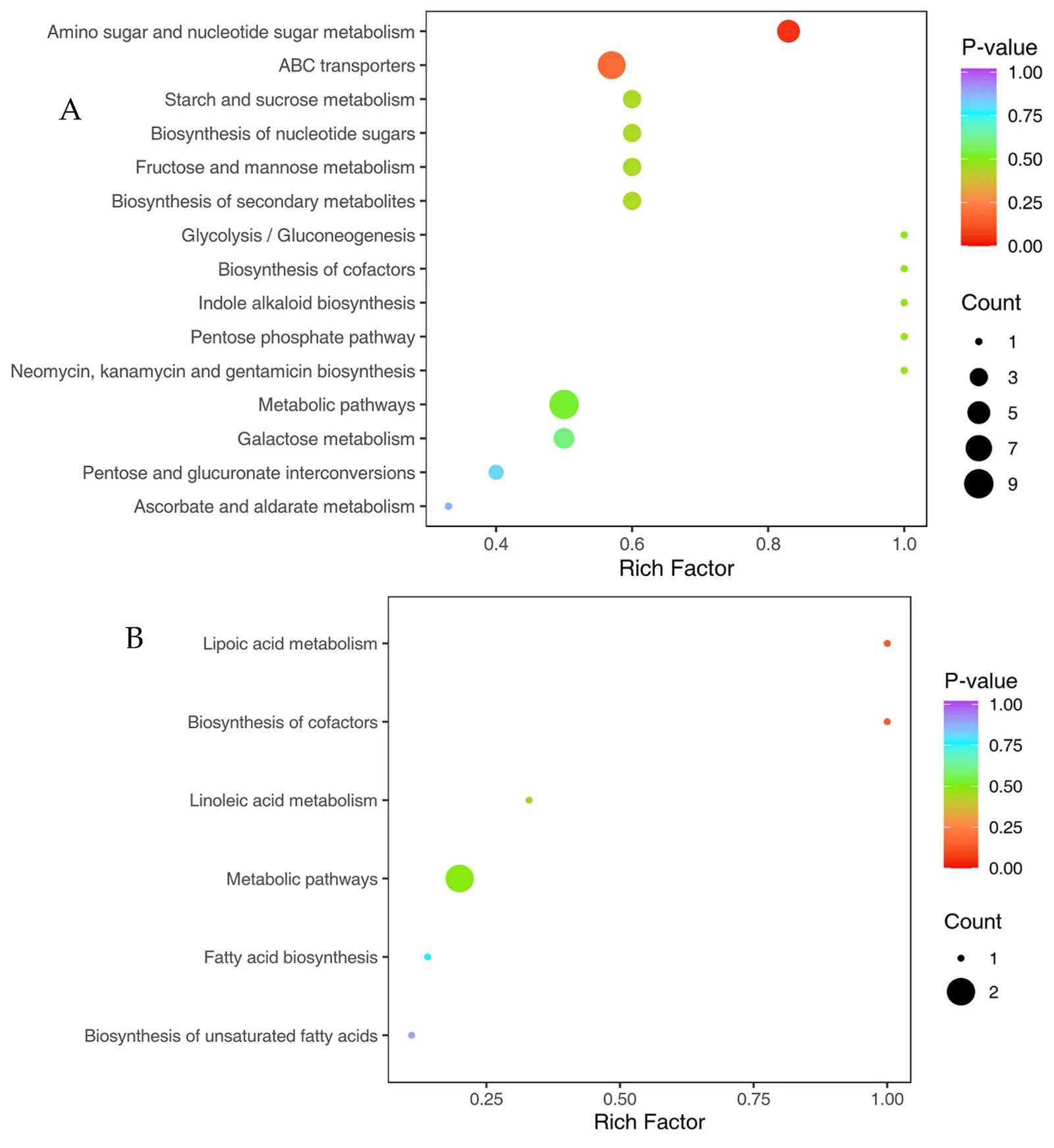
KEGG analysis of sugars and fatty acid metabolism at 7th day germination of seeds after two storage methods. (**A**) Sugar metabolism and (**B**) fatty acid metabolism.

To identify the major metabolic pathways involved in *X. sorbifolium* seed response to the two storage methods, we performed a KEGG metabolic pathway enrichment analysis on the resulting metabolites. As shown in Figure 9, and Tables S1 and S2, sugar metabolites are primarily enriched in amino sugars and nucleoside sugar metabolism (ko00520), ABC transporters (Ko02010), fructose and mannose metabolism (Ko00051), biosynthesis of nucleosides (Ko01250), and starch and sucrose metabolism (Ko00500) (Figure 9A, Table S1). Fatty acid metabolites are primarily enriched in linoleic acid metabolism (ko00591), biosynthesis of cofactors (ko01240), lipoic acid metabolism (ko00785), and unsaturated fatty acid biosynthesis (ko01040) and fatty acid biosynthesis (ko000061) pathways (Figure 9B and Table S2).

**Figure 10:**
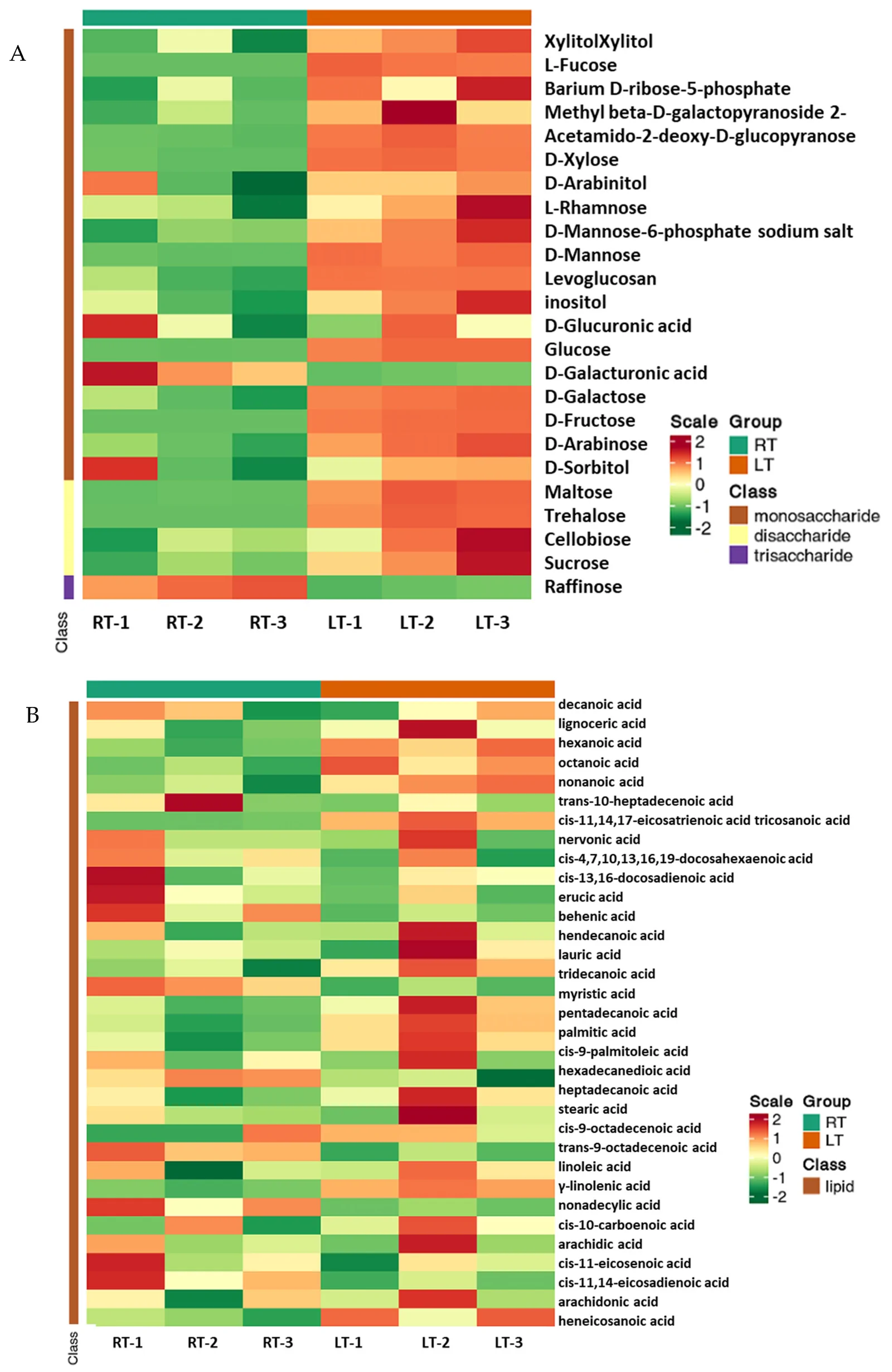
Heatmap of substance changes in sugars and fatty acid metabolism at 7th day of germination of seeds after two storage methods. (**A**) Saccharides and (**B**) fatty acid metabolism.

The standard equation for calculation and the GC-MS chromatogram for carbohydrate and fatty acid metabolites are shown in Tables S3 and S4, and Figure S2. A total of 24 sugar metabolites were detected (Figure 10A). Compared with storage at RT storage, 10 sugar metabolites showed significant differences, including eight that were upregulated (maltose, trehalose, L-fucose, N-acetylglucosamine, D-xylose, D-mannose, glucose, and D-fructose), with trehalose (Log_2_FC, 7.44), L-fucose (6.93), D-fructose (5.95), and glucose (4.87) showing the most pronounced upregulation; and two were downregulated (D-galacturonic acid and raffinose) (Table 2).

A total of 34 fatty acid metabolites were detected (Figure 10B). Differences in the expression levels of four fatty acids were observed between low-temperature refrigeration and room-temperature storage, with three showing upregulation (caprylic acid, cis-11,14,17-eicosatrienoic acid, and γ-linolenic acid) and one showing downregulation (nonadecanoic acid). Among these, cis-11,14,17-eicosatrienoic acid exhibited the highest Log_2_FC value, reaching 2.66 (Table 2).

**Figure 11:**
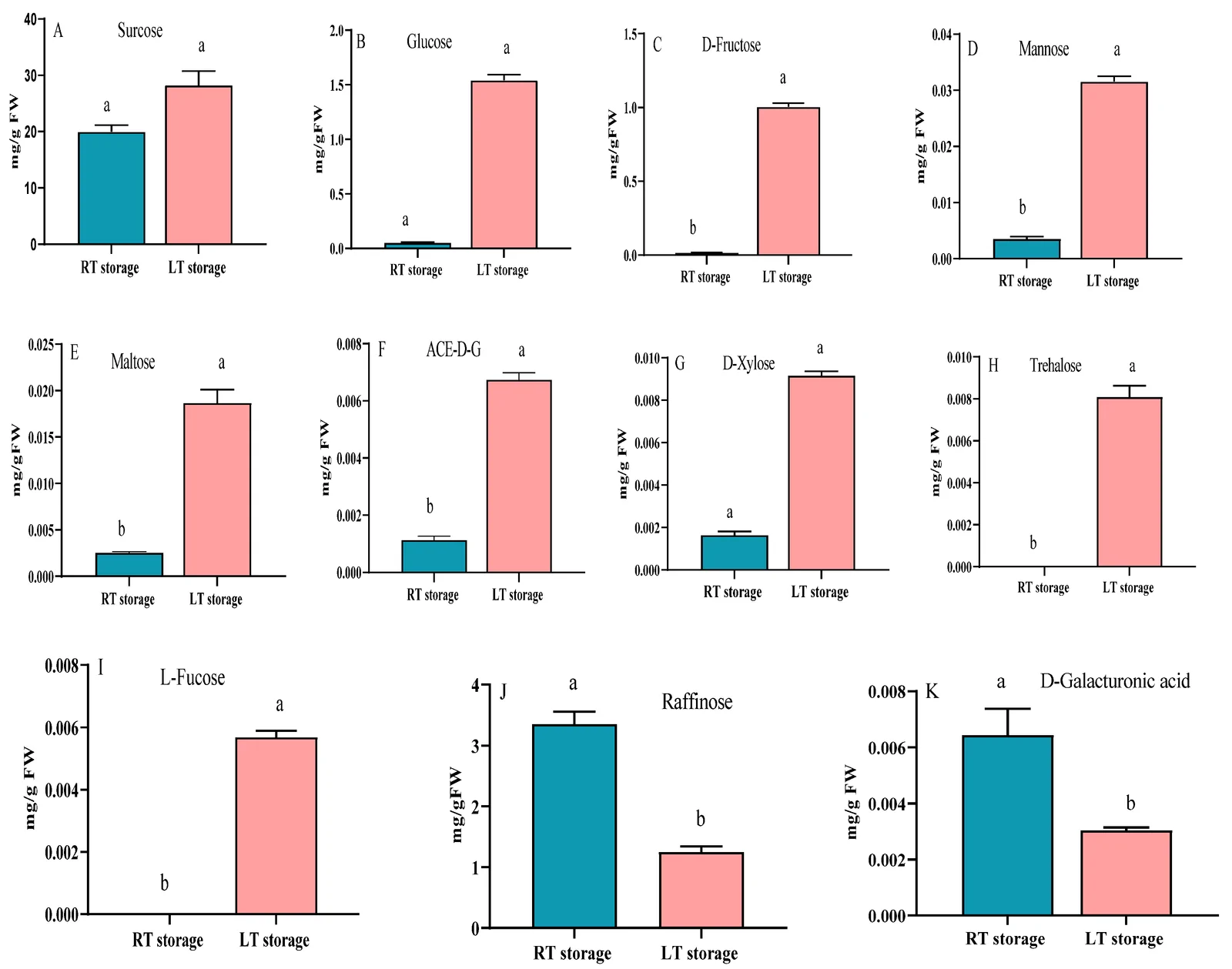
The content changes in differential metabolites in sugars metabolism at 7th day of germination of seeds after two storage methods. (**A**): Sucrose; (**B**): Glucose; (**C**): D-Fructose; (**D**): (D-Mannose; (**E**): Maltose; (**F**): ACE-D-G (acetamido-2-deoxy-D-glucopyranose); (**G**): D-Xylose; (**H**): Trehalose; (**I**): L-Fucose; (**J**): Raffinose; (**K**): D-Galacturonic acid. Data represent the mean ± SD of three biological replicates. Different lowercase letters indicate significant differences between two storage methods (*p* < 0.05).

Among of the significant increases in sugar contents, sucrose levels were highest in LT-treated of *X. sorbifolium* seeds, reaching 28.21 mg/g FW, a 41.2% increase compared to the RT-treated seeds. Glucose and fructose were the second-highest sugar compounds, with LT-treated seeds containing 1.54 mg/g and 1.00 mg/g FW, respectively. These values were 28.14 and 60.77 times higher than those in the RT-treated seeds, respectively. The third group of sugars with high concentrations were 2-Acetamido-2-deoxy-D-glucopyranose, maltose, D-mannose, and D-xylose, with their levels in the LT−treated being 4.92, 6.34, 7.90, and 4.59 times higher than those in the RT-treated seeds, respectively. Although the Log_2_FC values for trehalose and L-fucose were relatively high, RT-treated samples were not detected due to their extremely low concentrations. LT-treated samples had concentrations of 0.0081 mg/g FW and 0.0057 mg/g FW for trehalose and L-fucose, respectively. The two sugars that showed a significant decrease were raffinose and D-galacturonic acid, with concentrations in the LT treatment being 62.7% and 52.7% lower than in the RT treatment, respectively (Figure 11).

**Figure 12:**
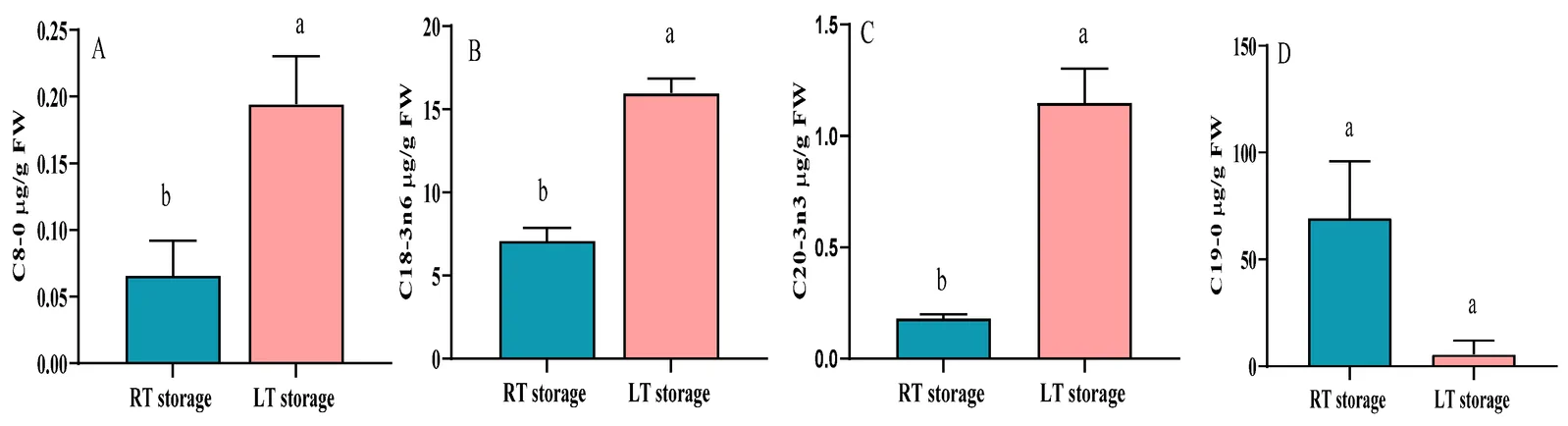
The content changes in differential metabolites in fatty acid metabolism at 7th day of germination of seeds after two storage methods. (**A**): C8-0 (octanoic acid); (**B**): C18-3n6 (γ-linolenic acid); (**C**): C20-3n3 (Cis-11,14,17-eicosatrienoic acid); (**D**): C19-0 (nonadecylic acid). Different lowercase letters indicate significant differences between two storage methods (*p* < 0.05).

Among the four fatty acids showing significant differences, stearic acid (C18-0) exhibited the highest content under LT treatment at 177.21 μg/g FW, which was 1.95 times higher than that under RT treatment. Under LT treatment, the contents of cis-11-γ-linolenic acid (C18-3n6) and 14,17-eicosatrienoic acid (C20-3n3) were 15.98 μg/g FW and 1.15 μg/g FW, respectively, representing increases of 1.25-fold and 5.32-fold compared to the RT treatment. In contrast, the content of nonadecylic acid (C19-0) decreased, with the LT−treated sample showing a 91.7% reduction compared to the RT−treated sample (Figure 12).

### 2.8. Analysis of the Correlation Between Fatty Acids, Sugar Metabolites, and Physiological Indicators

**Figure 13:**
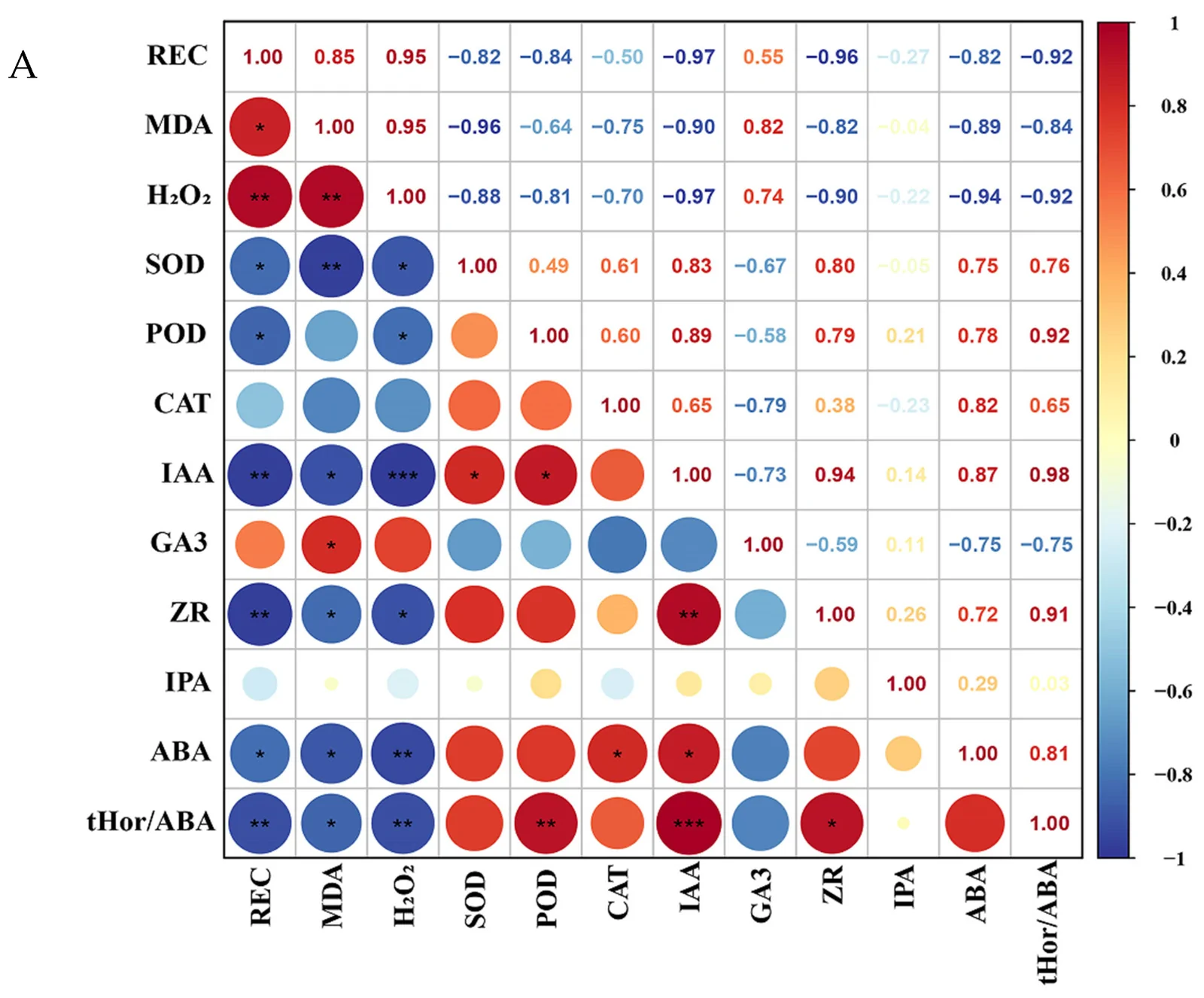

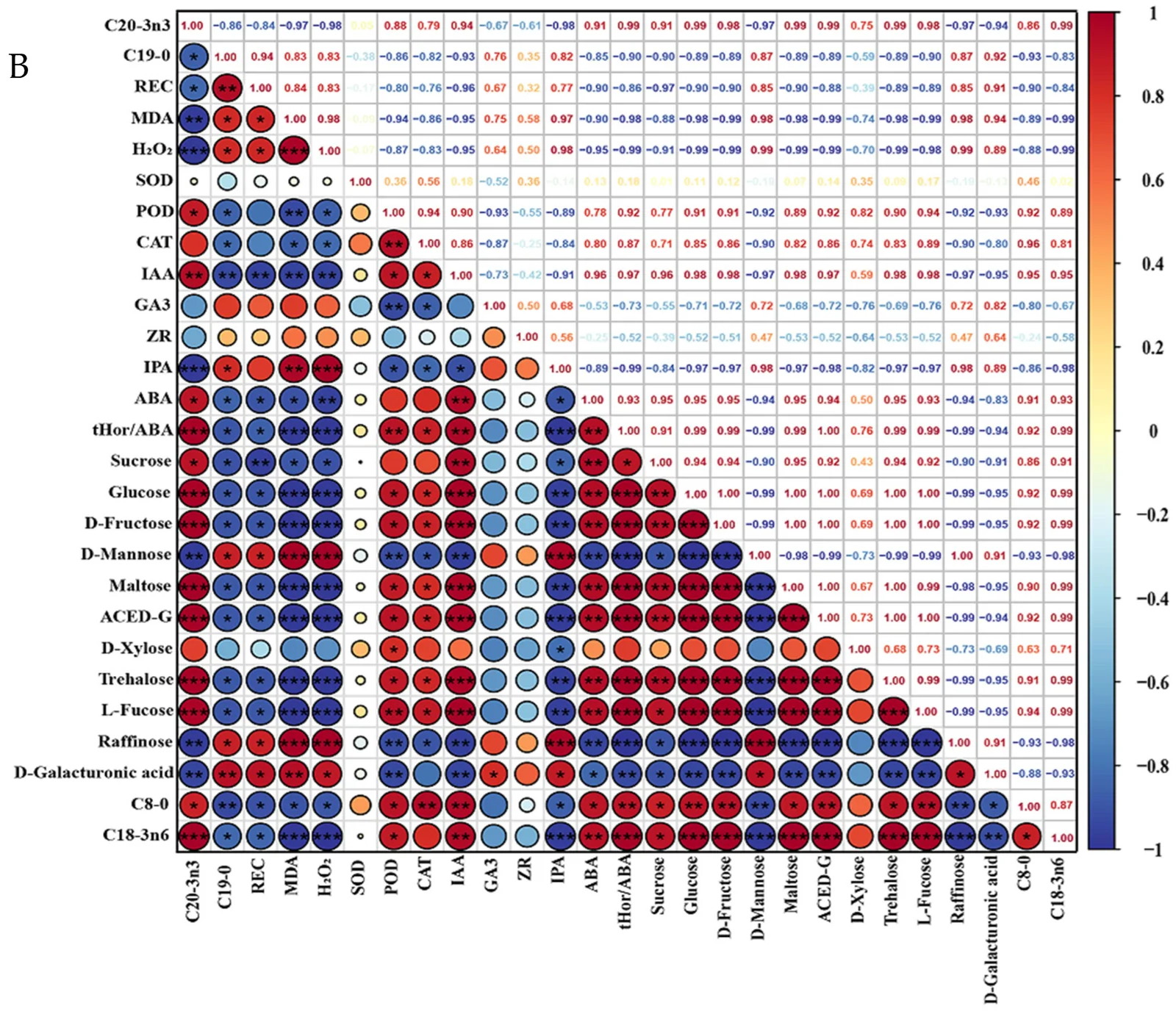
Heat map of correlation between metabolites and physiological indicators on day 0 of germination (VI stage) (**A**) and on the 7th day of germination of seeds (VII stage) (**B**) after RT and LT storage. ACED-G, 2-Ace-2-Deo-D-Glucosamine; C8-0, octanoic acid; C20-3n3, Cis-11,14,17-eicosatrienoic acid; C18-3n6, γ-linolenic acid; C19-0, nonadecylic acid. * *p* < 0.05; ** *p* < 0.01; *** *p* < 0.001. The colors of these circles are determined by the significance data size shown in the rectangle graph on the right. The darker the red, the stronger the positive correlation; the darker the blue, the stronger the negative correlation.

Pearson’s correlation analysis was conducted between the antioxidant system parameters and hormones on day 0 of germination (VI) (Figure 13A), and between the metabolites such as sugars and fatty acids, and the parameters such as antioxidant system and hormones, on the 7th day of seed germination (VII) (Figure 13B). An extremely significant negative correlation was found between three parameters, namely H_2_O_2_, MDA, and REC, and six parameters, namely SOD, POD, IAA, ZR, ABA, and tHor/ABA (*p* < 0.05, *p* < 0.01 or *p* < 0.001), except between POD and MDA. Conversely, an extremely significant positive correlation was found between SOD and IAA; between POD and tHor/ABA or IAA; between CAT and ABA, and between IAA and tHor/ABA, ABA or ZR; between ZR and IAA (*p* < 0.05, *p* < 0.01, or *p* < 0.001) during the VI period (Figure 13A).

On the 7th day of germination (VII), there was an extremely significant (*p* < 0.01; *p*< 0.001) or significant (*p* < 0.05) negative correlation between the H_2_O_2_, MDA, REC, and C19-0 indicators and the C18-3n6, C8-0, Cn20-3n3, trehalose, L-fucose, ACED-G, maltose, glucose, D-fructose, sucrose, ABA and tHor/ABA, IAA, POD, and CAT indicators, except between REC and POD or CAT. However, an extremely significant or significant positive correlation was observed between the H_2_O_2_, MDA, REC, and C19-0 indicators and the D-mannose, raffinose, D-galacturonic acid, and IPA indicators, except between REC and IPA. Similarly, an extremely significant or significant positive correlation was observed between the POD and CAT indicators, and the C8-0, D-xylose, trehalose, L-fucose, ACED-G, maltose, glucose, D-fructose, tHor/ABA, and IAA indicators, except between CAT and D-xylose or C18-3n6. In addition, there was an extremely significant or significant positive correlation between the ABA, tHor/ABA, and IAA indicators and the C18-3n6, C8-0, Cn20-3n3, trehalose, L-fucose, ACED-G, maltose, glucose, and sucrose indicators. Conversely, there was an extremely significant or significant negative correlation between the ABA, tHor/ABA, and IAA indicators and the D-mannose, raffinose, and D-galacturonic acid indicators (*p* < 0.05 or *p* < 0.01) (Figure 13B).

## 3. Discussion

*X. sorbifolium* has gradually attracted worldwide attention due to its excellent economic and ecological value as a woody oil and ornamental tree species [40]. However, the seeds of *X. sorbifolium* exhibited dormancy characteristics and have a low germination rate. Unfortunately, knowledge of the potential mechanisms of dormancy release and germination in *X. sorbifolium* is limited. In our work, the germination rate of seeds stored at LT condition was higher 44.9% than that of seeds stored at room temperature (RT). Other indicators, such as germination vigor, germination index, and seedling growth, were all optimal. Some researchers have also reported low-temperature storage could enhance the seed germination. For example, the germination rate of *Swertia chirayita* seeds stored at −15 °C was 60.0% compared to 48% storing at 25 °C after 18 months of storage [41]. Seed viability was maintained at over 90% for three months at −20 °C in *Morinda royoc* seeds [42]. Our previous work also found that the ‘Qihong’ variety of *X. sorbifolium* had a germination rate of 41.3% in RT condition, but this increased to 58.3% after 60 days of LT storage. Moreover, the seed germination rates peaked after 60 days of LT storage, and then they decreased when the storage period was extended to 90–150 days [2]. Furthermore, previous studies have also confirmed that the germination rate of *X. sorbifolium* increased at −20 °C [35,36,37,38]. This suggests that the *X. sorbifolium* seed dormancy release might require low temperatures. Low temperature could act as an external signal, promoting changes in gene expression, enzymes, and metabolites within the seeds, and breaking the seed dormancy. Our work also found that the method of direct germination in moist sand (GMS) after storage was better than other methods, such as PEG + GMS, 35 °C + GMS and ABT + GMS. Therefore, the optimal method for breaking seed dormancy and promoting the germination of *X. sorbifolium* seeds was −20 °C low-temperature storage (LT) for 60 days, followed by germination in moist sand.

The hardness and thickness of the seed coat (shell) affect water permeability, leading to physical dormancy. The presence of macrosclereids in the palisade layer of *Amazonian mimosa* L. seeds decreased the seed coat impermeability and led to physical dormancy [43]. In *Taxus yunnanensis* seeds, the seed coat was observed to have larger and numerous cracks, and a severely damaged surface layer after storage for 30–360 days; this made it easier for water to enter the seed and promote the germination [44]. Wheat seeds were also seriously damaged, with more holes caused by the abscission observed in storage at high moisture and room temperature [45]. The seed shell of *X. sorbifolium* is relatively hard, and it might also undergo physical dormancy. However, in our work, the seed shells and kernels of *X. sorbifolium* stored at room temperature (RT) exhibited symptoms such as wrinkling, cracking, and roughness. In contrast, the shells and kernels remained intact and smooth when stored at low temperature (LT) under SEM observation (Figure 4). This indicated that the seeds stored at RT condition suffered shell and kernel damage. If *X. sorbifolium* seed dormancy is primarily due to the hardness of the seed shell, the damage of seed shell should enhance the seed’s ability to absorb water and improve the seed germination rate. Our data about the moisture content (MC) and water absorption rate (WAR) of seeds verified that the water-absorb content was higher for RT-stored seeds than for LT-stored seeds during the early germination period. However, the various germination indicators of the seeds and the growth conditions of the seedlings all proved that the seeds subjected to RT condition ware significantly worse compared to those stored at LT condition (Figure 2 and Figure 3; Table 1). This indicated the increased water absorption of RT-stored seeds was caused by damage to their shells, resulting in passive water absorption. Nevertheless, this did not lead to an increase in the seed germination rate. Furthermore, we simultaneously observed that the seed kernels were damaged; this was the reason why the germination rate of RT-stored seeds was low. Therefore, the physical dormancy (i.e., the hardness and thickness of seed shell) might affect the seed germination, but it was not the main cause of dormancy in *X. sorbifolium* seeds. Physiological dormancy (the vitality of the seed kernels) might be the primarily reason, as maintaining kernel integrity and awakening kernels were the main condition for enhancing seed germination.

Many researches have confirmed that low-temperature storage could promote seed germination. Miscanthus seeds exhibited the highest germination rate and vitality index, sustaining only minor structural damage when stored at –18 °C with desiccant. In contrast, germination rates dropped, and the seeds lost nearly all their vitality under room-temperature storage conditions [46]. Similarly, Gu et al. (2018) [47] found that the germination rate of *Ruppia sinensis* seeds was higher at −10 °C storage than that at 0 °C storage for a week. Wheat and barley seeds showed high germination rates of 94% and 90%, respectively, when stored at −20 °C for 23–33 years [48]. However, the opposite result was found in *Carissa edulis* and *Ximenia americana* seeds, for which low seed moisture content and temperature (−20 °C) reduced seed viability [49]; this might be due to their being tropical species, and their seeds ware not tolerant of low temperatures. In our study, *X. sorbifolium* seeds stored at low temperatures remained intact and had plump kernels during the early stages of germination, exhibiting a slower water uptake rate than those at RT condition. In addition, the method of direct moist-sand germination (GMS) might prevent the endosperm (kernels) of seed from sustaining physical damage due to excessive swelling during the initial stages.

It has been confirmed that ROS are involved in the regulation of seed dormancy release and germination. Excessive ROS accumulation would cause oxidative damage and inhibit seed germination, and even lead to seed deterioration [26,50,51]. Therefore, maintaining ROS homeostasis was essential for the release of seed dormancy and germination. This suggested that seed germination was likely to occur only when ROS levels were within the ROS signaling range rather than the ROS damage range [52]. In our work, the H_2_O_2_ content increased significantly at the end of the seed storage period (VI) and the 7th day of germination (VII) following storage at room temperature. Similarly, the MDA content was significantly elevated at the same two periods. In addition, the RECs of the seed shells and kernels were also significantly higher at stage VI after RT storage. Meanwhile, severe damage to the seed kernels was observed at the same two stages (VI and VII) after RT storage. Our results are in agreement with those of other researches. The H_2_O_2_ and MDA levels in Pyrenes seeds increased by 5.7-fold from 60 to 365 days during storage at 17.8 °C and 35 °C, and were significantly higher than in freshly dispersed seeds [53]. This illustrated that inappropriate RT storage led to excessive ROS accumulation, which then caused oxidative damage and seed deterioration. This might explain why the seeds have a low germination rate when stored at room temperature.

In contrast, the levels of H_2_O_2_, MDA, and REC of *X. sorbifolium* seeds all significantly decreased at the same two stages (VI and VII) in the LT condition. Meanwhlie, the SOD, CAT, and POD activities were significantly higher under the LT condition than under the RT condition, especially on the 7th day of germination (VII). These findings ware consistent with those reported by Zhang et al. (2022) [54], who found that low-temperature stratification increased POD activity, which was beneficial for breaking the dormancy of *Phoebe hui* seeds. During germination, the MDA and H_2_O_2_ content of cotton seeds decreased significantly, while SOD activity increased after GA treatment at a low temperature (12–15 °C), compared to storage at 25–28 °C [55]. Similarly, the MDA content of pear stock seeds increased significantly at room-temperature storage, while SOD, CAT, and POD activities were higher in seeds stored at 4 °C than those stored at room temperature [56]. Antioxidant activity also increased after chilling storage, and improved the germination percentages (from 46% at room temperature to 100% at −75 °C) in *Geum urbanum* seeds [57]. These indicated seeds were subjected to oxidative stress at room temperature. Conversely, low-temperature preservation maintained ROS levels within the signaling range and facilitated the gradual release of seed dormancy. Additionally, the direct moist-sand condition (GMS) also enabled the *X. sorbifolium* seeds to maintain an appropriate level of ROS during germination.

Plant hormones, especially gibberellins (GAs) and abscisic acid (ABA), are considered key factors in the regulation of seed dormancy and germination [18,58,59]. This regulation is determined by the level of the ABA/GA ratio; a low ratio promotes seed germination [24]. Other hormones, such as auxin (IAA), cytokinin, zeatin riboside (ZR), and isopentenyladenine (IPA), could also regulate seed dormancy and germination synergistically, involving cross-talk through the ABA or GA signaling pathways [60]. For example, the ratios of GA1, 7, 12, and 20/ABA and IAA/ABA increased significantly in rice seeds during storage to germination, while GA3 and 4/ABA and GAs/IAA ratios decreased significantly [61]. During 360 days of wet sand storage, the GA and ZT (zeatin riboside) content of *Taxus yunnanensis* seeds remained relatively stable. However, while the IAA content and the IAA/ABA ratio increased, the ABA content decreased as the storage time was extended [44]. The ABA content of sunflower seeds was inhibited at a low temperature (−20 °C) for 12 h, whereas it increased when stored at room temperature for 33 days [60]. The levels of ABA were observed to decrease markedly, while GA3 increased, after a 60-day cold stratification period, compared to 0 days (i.e., before cold stratification) [62]. In addition, the contents of ZR, IAA, and GA in the embryos were higher under low-temperature (4 °C) storage for 27 days than room storage, while the ABA levels decreased in *Paeonia suffruticosa* seeds [63].

In our work, the ABA content decreased by 23.2% on the 7th day of germination (VII) in the LT treatment compared to the RT treatment; this was contrary to the result of increased H_2_O_2_ content during the same period. Other reports have also found ROS enhanced, while ABA degradation and GA increased in rice (*Oryza sativa*) or in *Smilax glabra* during seed storage and germination [14,29]. This indicated that the ROS and the hormone signals had undergone cross-talk and were jointly promoting seed germination. It was verified that ROS in signal level (especially H_2_O_2_) could enhance the release of seed dormancy by inducing the expression of ABA 8′-hydroxylase gene (*CYP707A*, a key enzyme in ABA catabolism), thereby promoting ABA degradation. This illustrated that the signaling pathways of ROS and ABA ware mutually antagonistic. ROS promoted the degradation of E3 ligases and PYR/PYL receptors related to ABA, thereby restraining ABA signaling and enhancing germination [27,64]. However, ROS could also stimulate GA biosynthesis by modulating the expression of *GA3OX1*, a GA-related biosynthetic gene, thereby promoting seed germination. This indicated that there was a synergistic relationship between the ROS and GA signaling pathways [65], and this synergy was achieved by ROS simplifying the degradation of the DELLA protein and releasing the GA response genes [27]. In addition, GA could decrease ABA levels by inhibiting *NCED6* expression and enhancing *ABI4* degradation (an ABA degradation relation gene) [66], and regulate seed dormancy and germination.

The difference in our work was a slight decrease in GA3 content in the LT condition, and there was no significant deference between the two storage methods. However, the contents of IAA, IPA, and ZR, and the ratios of IAA/ABA and (IAA + GA3 + ZR + IPA)/ABA (tHor/ABA) increased significantly, especially the tHor/ABA ratio, which increased by 62.7% at stage VII under LT condition compared to the RT condition at the same stage, while the GA/ABA ratio was only significantly higher under the LT condition at stage VII. This suggested that IAA and cytokinins (ZR + IPA) also play important roles in seed dormancy and germination. Many reports confirmed that IAA repressed seed germination in conjunction with ABA during seed dormancy [67,68]. For example, exogenous IAA has been shown to promote ABA biosynthesis and reduce GA levels, thereby inducing seed dormancy in *Glycine max* [69]. During imbibition of *Arabidopsis thaliana* seeds, IAA levels were found to be increasing, while ABA levels were reducing [70]. This suggested that ABA restrains seed germination via the auxin signaling pathway. Furthermore, IAA promoted ABA signaling by enhancing the expression of *ARF10* and *ARF16*, which acted upstream of *ABI3*. Meanwhile, *ABI5*, which acted downstream of *ABI3*, mediated the inhibition of ABA on seed germination [66,67].

**Figure 14:**
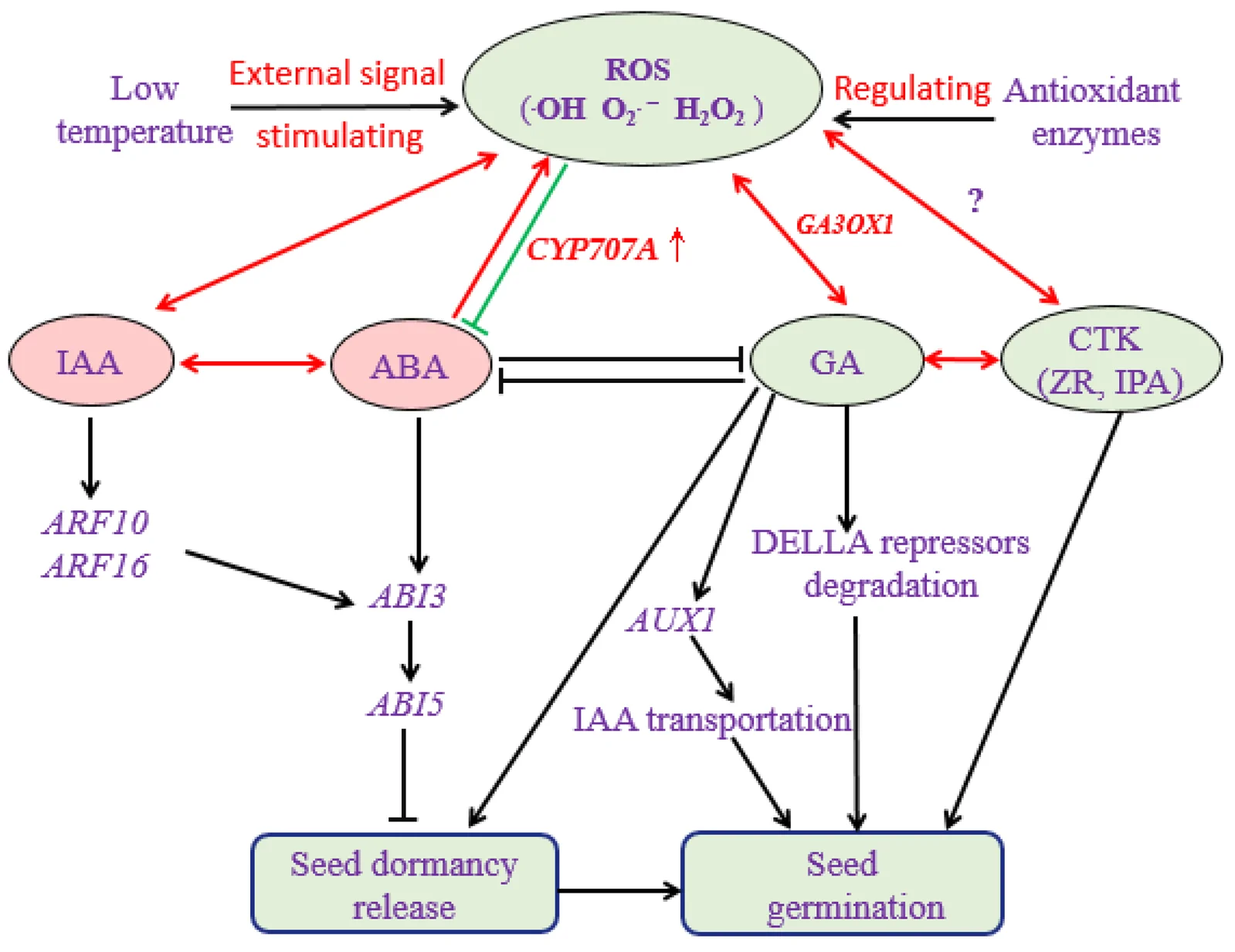
The cross-talk between ROS-GA-ABA-IAA under low-temperature storage during seed dormancy release and germination. ↑: up regulation; 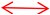: synergistic relationship; 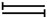: mutually antagonistic relationship; 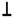: repress.

However, during seed germination, IAA is redistributed into seed compartments via IAA transport, and *AUX1* facilitates the delivery of IAA to the radicle tip to promote germination [71]. This indicates that targeted transportation of IAA to embryonic tissues is a modulatory step following seed dormancy release. During this progress, IAA promoted seed germination in conjunction with GA and counteracted the inhibitory effect of ABA [68]. Therefore, during the seed dormancy release and germination, cross-talk occurs among signals from ABA, GA, IAA, and ROS, which jointly regulate seed germination. This was achieved through the continuous changes in the ratio between hormones or ROS. The IAA/ABA and tHor/ABA ratios were more important than the ratio of GA/ABA to seed dormancy and germination in *X. sorbifolium* seeds. Higher IAA/ABA and tHor/ABA ratios, rather than individual GA/ABA, are more beneficial for seed dormancy release and germination. The cross-talk between ROS−GA−ABA−IAA is shown in Figure 14.

In addition to hormones and reactive oxygen species, the metabolism of sugars and lipids also plays a key role in influencing seed dormancy and germination in plants [72], especially the content of sugars and lipids and the composition of fatty acid in oilseed seeds [2,73]. During the storage of *Lilium pumilum* bulbs at 4 °C for 90 days, transcriptome data indicated that carbohydrate metabolism was activated to break dormancy [74]. In the *Smilax glabra* seed dormancy-release process, the soluble proteins and starch content decreased, while the expression of starch hydrolase genes (AMY and BAM) were significantly upregulated, and this facilitated starch being degraded into soluble sugars and broke seed dormancy [14]. Multiple enzymes genes involved in starch degradation, such as starch phosphorylase, 4-alpha-glucanotransferase, and beta-glucosidase, exhibited expression upregulation during dormancy release through cold stratification in *Cercis chinensis* seeds [75]. Metabolomic analysis indicated the levels of phenolic compounds and carbohydrates, such as antifreezing sugar alcohols (predominantly threitol), and defense-related metabolites (1,2,4-Benzenetriol or BTO) and ascorbic acid degradation increased with reduction in the germination capacity of *Quercus robur* seeds stored at −7 °C for six months [76]. Storage mature seeds of *Libidibia ferrea* at −18 °C for six months maintained the proportions of the tricarboxylic acid cycle, such as citric, malic acids, methyl-inositol, and xylitol, while the proportion of quinic acid and myo-inositol increased, indicating a metabolic switch during seed storage [39]. After being stored for 60–300 d at −18 ± 2 °C, *Sinojackia xylocarpa* seeds contained more soluble carbohydrates, such as D-xylose and sucrose, upregulated compared to storage at 4 ± 2 °C [77]. In our work, the changes in carbohydrate and lipid metabolites were analyzed using targeted metabolomics during the germination of *X. sorbifolium* seeds after −20 °C storage. There are 10 differential metabolites (DMs) of the 24 carbohydrate compounds, with eight showing upregulation and two showing downregulation. Among these, glucose, D-fructose, trehalose, and L-fucose exhibited the greatest upregulation; the contents of sucrose, glucose, and fructose were especially higher than others. Based on our previous research [2], from the end of LT storage (stage VI) to the 7th day of seed germination (stage VII), the fat content of *X. sorbifolium* seeds decreased from 59.5% to 54.4%, while the starch content decreased from 1.6% before storage to 0.9%. This indicated that low-temperature storage, combined with the moist-sand treatment, led to the degradation of starch in the *X. sorbifolium* seeds, and a large amount of soluble sugars provided energy for the newly formed radicle and plumule, thereby promoting germination.

During the germination of oilseeds, lipids are degraded into fatty acids through β-oxidation, and then transformed into carbohydrates via the glyoxylate cycle; this provides energy and carbon skeletons for seed development. This process is accompanied by the massive production of ROS and lipid peroxidation [27]. For instance, the lipid oxidation of pumpkin-seed kernels was significantly lower under refrigerated storage conditions than at room temperature. In addition, total lipid levels, including lysophosphatidic acid (LPA), monoglyceride, triglycerides, phosphatidylcholine (PC), and phosphatidylethanolamine (PE), increased significantly under refrigerated storage compared to room-temperature storage [25]. Free fatty acids (FFAs), such as FFA(18:0), FFA(20:0), FFA(18:1), and FFA(18:2), in *Sinojackia xylocarpa* seeds were significantly increased at −18 ± 2 °C storage compared to at 4 ± 2 °C storage for 60–300 days [77]. In our work, we identified 34 fatty acid metabolites during *X. sorbifolium* seed germination. Among these, caprylic acid (C8-0), eicosatrienoic acid (C20-3n3), and γ-linolenic acid (C18-3n6) were significantly upregulated after low-temperature storage, while nonadecanoic acid (C19-0) was downregulated. Our findings are consistent with the previous researches’ results showing that the soluble sugars and unsaturated fatty acids increase during seed storage. The increase in unsaturated fatty acids such as γ-linolenic acid was beneficial for enhancing the antioxidant capacity during seed germination.

In short, the integrity of *X. sorbifolium* seeds was damaged during storage at room temperature (RT), affecting both the shell and the kernel. The damaged kernels resulted in excessive water uptake and H_2_O_2_ accumulation, as well as a decrease in protective enzyme activity during germination. Consequently, the seeds were unable to maintain the appropriate ROS levels for germination. Additionally, the high ABA level made it difficult to break seed dormancy. This led to accelerated seed deterioration, and a low germination rate.

**Figure 15:**
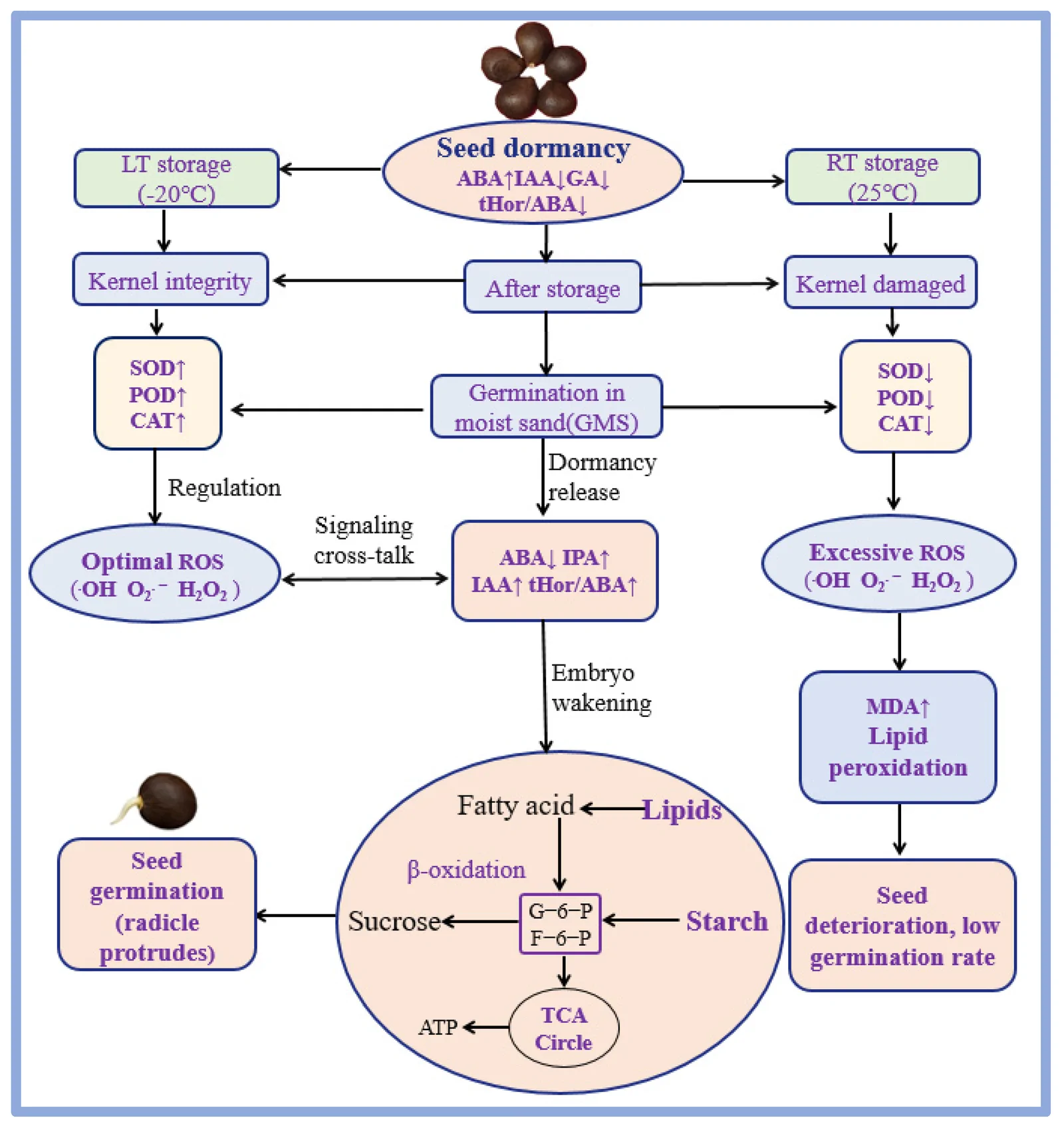
The mechanism by which low-temperature storage promoted the seed dormancy release and germination in *X. sorbifolium*. ↑: increase; ↓: decease.

Low-temperature (LT) storage preserved the high integrity of the seed shells and kernels—particularly the kernels. This could maintain high activities of protective enzymes, reduce ABA content, and increase the levels of tHor/ABA and IAA/ABA ratios, as well as IAA, and kept ROS within signal-transduction levels. This contributed to cross-talk of ROS−GA−ABA−IAA signaling pathways. The interactions were beneficial for the coordinated regulation of the kernels wakening process, allowing the seeds to gradually emerge from dormancy and reach a state ready for germination. In addition, germination in moist sand (GMS) created a suitable microenvironment in which starch decomposed into soluble sugars and lipids decomposed into fatty acids. These fatty acids were then converted into soluble sugars, which provided energy and carbon skeletons for seed development, stimulating vigorous seed metabolism and improving the germination rate. Figure 15 shows the mechanism by which low-temperature storage promotes the seed dormancy release and germination in *X. sorbifolium*.

## 4. Materials and Methods

### 4.1. Plant Materials and Seed Storage Methods

*X. sorbifolium* seeds were obtained in November from the germplasm resource garden of *X. sorbifolium* (Shandong Woqi Agricultural Development Co., Ltd., Weifang, China).

The seeds were first soaked in a 0.3% KMnO_4_ (potassium permanganate) solution for 15 min, then rinsed thoroughly with tap water, and dried in a cool, shaded place before storage. The seeds were placed randomly in a perforated, sealable plastic bag and stored at a low temperature (LT) in a desiccant at −20 ± 0.5 °C for 60 days. A control group was stored at room temperature (RT) (25 ± 0.5 °C) in desiccant condition for 60 days. Both LT and RT groups consisted of 1200 seeds each (100 seeds per bag).

### 4.2. Seed Germination Treatments and Seedlings Planting

After 60 days of storage, the seeds were used for following germination methods: (1) direct germination in moist sand (GMS) (uniformly sized grains used the 121 °C autoclaved sterilized) after storage; (2) after storage, the seeds first soaked in water at 35 °C for 5 days, then germinated (35 °C + GMS); (3) after storage, the seeds were first soaked in 200 mg/L ABT for 5 days, then germinated (ABT + GMS); and (4) after storage, the seeds were first soaked in 5% PEG for 5 days and then germinated (PEG + GMS).

The seeds were placed in the germination box with moist sand (sterilized sand mixed with water) after storage. The standard for the moist sand was that the mixture could form a clump when squeezed in the hand but could fall apart upon release, reaching a moisture content of approximately 60%. Place 100 seeds in each germination box (with 3–4 times seed volume of moist sand), and cover the top with a 3 cm layer of moist sand. The boxes were then placed in a dark at room temperature to germinate. There were three germination boxes for each treatment (three replicates). During the germination, turn the seeds periodically to ensure proper aeration, and spray regularly with water to maintain consistent humidity. Observe and record the number of germinated seeds daily for 28 days.

Transplant the germinated seeds into a growing medium with a mixture of peat soil, perlite, and vermicompost (in a 2:1:1 volume ratio), to which 0.1% carbendazim has been added. Use one 10 cm × 8 cm non-woven fabric bag per seedling for cultivation. Apply 1/4 MS nutrient solution watered once a week. The survival rate and growth parameters will be determined 30 days after transplanting. Growth indicators such as germination rate, survival rate, seedling height, main root length, and fresh weight (FW) and drought weights (DW) of seedlings were determined on the 28th day after germination or transplanting.

Germination rate (%) = Total of germinated seeds/total seeds tested × 100%.

Germination potential (%) = Seeds of germinated during 14 days/Total number of seeds tested) × 100%.

Germination Index (GI) = ∑(Gt/Dt)

Gt, germinated seeds within the time t; Dt, the number of germination days within the time t; and ∑, the sum Gt/Dt.

Survival rate (%) = Surviving seedlings/total transplanting seedlings × 100%.

### 4.3. Research on the Mechanism of Seed Dormancy Release and Germination

**Figure 16:**
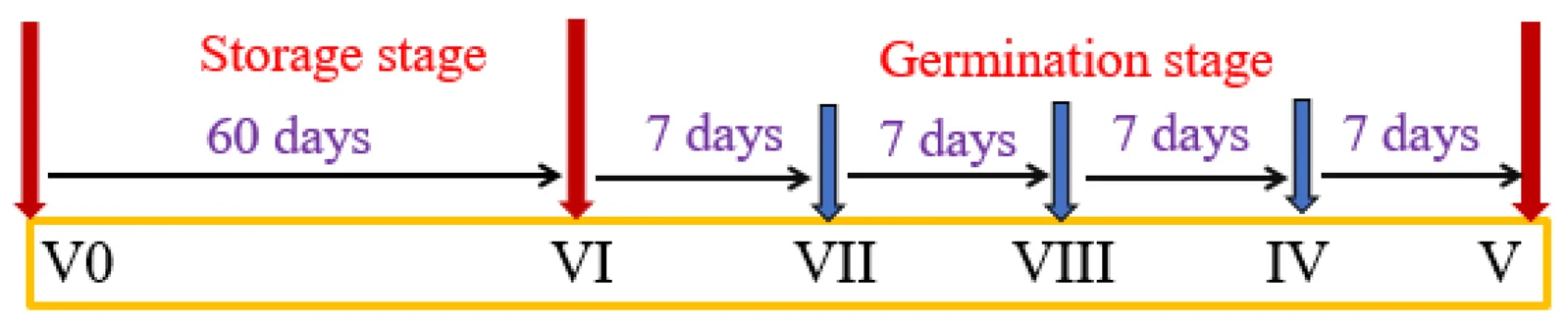
The six sampling time periods during seed storage and germination process of *X. sorbifolium*.

The seed storage methods (LT and RT) and germination treatment (GMS) were the same as described above. There were 1200 seeds in each of the LT and RT groups (100 seeds per bag). After 60 days of storage under RT and LT conditions, the seeds were germinated using the GMS method. Three boxes, each containing 20 seeds, were used to determine the water absorption rate. Six boxes, each containing 100 seeds, were used to determine the moisture content and electrical conductivity. Remaining seeds were used for enzymes, hormones, SEM, and metabolomics analysis. The remaining seeds were divided into 6 sampling time periods: before storage (stage V0), at the end of the storage (that also is the beginning direct germination in moist sand after storage) (stage VI), on the 7th day of moist-sand germination (stage VII), on the 14th day of moist-sand germination (stage VIII), on the 21th day of moist-sand germination (stage IV), and on the 28th day of moist-sand germination (stage V) (Figure 16). The above-mentioned indicator measurements were all repeated three times.

#### 4.3.1. Seed Water Absorption, Moisture Content, and Relative Electric Conductivity Analysis

There was a total of 8 sampling points for seed water absorption rate (WAR), moisture content (MC), and relative electric conductivity (REC) determination, including stage VI, and during the first seven days of the seed germination process. Samples were taken every 8 h for the first 72 h, and then every 24 h between 72 and 168 h.

To determine the water absorption rate, 20 seeds from each treatment were placed in a moist germination box to germinate. At each sampling point, the seeds were removed, washed, and dried with filter paper. After measured the fresh weight, they were returned to the germination box to continue germinating. At the second sampling point, the seeds were also removed and weighed again, and so on, until the eighth weighing. To determine MC, taking 10 seeds at each sampling time, after weighing the fresh weight (FW), we placed the seeds at 105 °C for 15 min, followed at 80 °C until constant weight (DW). After taking 10 seeds at each sampling time, we separated them into shells and kernels for REC determination. All of the above-mentioned indicator measurements were repeated three times.

The REC determination followed Chen’s method (2014) [78]. The seed shells and kernels were soaked in ultrapure water at room temperature for 1 h, and then we measured the electrical conductivity using an electrical conductivity meter (DDS-307A, Shanghai Inesa Scientific Instrument Co., Ltd., Shanghai, China). The REC unit was μS/cm (S: siemens).

WAR (%) = (The fresh weight at a later time point (FWn + 1) − The fresh weight at an earlier time point (FWn))/the fresh weight before germination (FW0), (n from 1 to 8, n: represents the sampling time point number)

Seeds MC (%) = (FW − DW)/FW × 100.

#### 4.3.2. Scanning Electron Microscopy Analysis

Take 10 seeds each from LT and RT groups at stages VI and VII, separate the seed shells from the kernels, and prepare them for scanning electron microscopy (SEM) (JSM-7600 F, JEOL Ltd., Tokyo, Japan) according to the previous methods [78].

#### 4.3.3. Assay of Antioxidant Enzymes and Hormones

The seeds from each germination period were sampled. The seeds were macerated in liquid nitrogen, and then stored at −80 °C to determinate the following parameters and to perform metabolomic analyses. Samples of SOD, POD, and CAT, as well as H_2_O_2_ and MDA, were measured in 6 stages, while samples of IAA, GA3, ZR, IPA, and ABA were determined at stages V0, VI, VII, and V. These seeds were removed from their shells when determining.

The measurements of SOD, POD, CAT, H_2_O_2_, and MDA were conducted according to the method of Feng et al. (2025) and Chen et al. (2018) [32,79].

The contents of five plant hormones, namely indoleacetic acid (IAA), gibberellin (GA), Zeatin-riboside (ZR), isopentenyladenine (IPA), and abscisic acid (ABA), were determined by China Agricultural University using the enzyme-linked immunosorbent assay (ELISA), following Teng et al. (2006) [80] and Yang et al. (2001) [81].

The samples were homogenized in liquid nitrogen and extracted in cold 80% (*v*/*v*) methanol with 1 mmol·L^−1^ BTH (butylated hydroxytoluene) at 4 °C for 12 h. We collected the extracts after centrifugation at 10,000× *g* (4 °C) for 20 min, and then passed them through a C18 Sep-Pak cartridge (Waters, Milford, MA, USA) and dried in N2. The residues were used for ELISA determination. The absorbance was measured at 490 nm using an ELISA Recorder (EL310, Bio-TEK, Winooski, VT, USA). In this work, the percentage recovery of each hormone was calculated by adding a known quantity of the standard hormone to a split extract. Among five hormones, the cross-reactivity of IAA antibodies with NAA is 16.7%, with a percentage recovery rate of 95.1%. The percentage recovery rate of GA and IPA was 89.1% and 90.2%. Cross-reactivity of the ZR antibodies with Zeatin is 47%, and percentage recovery rate was 86.1%. All of the sample extract dilution curves were paralleled to the standard curves, indicating the absence of non-specific inhibitors in the extracts.

#### 4.3.4. Targeted Fatty Acid and Sugar Metabolomics Analysis

Seeds at day 7 of germination (stage VII) were used for the metabolomics analysis, with a total of 6 samples. RT indicates that seeds were stored at room temperature for 60 days and then subjected to moist-sand germination on day 7 (3 replicates); LT indicates that seeds were stored at −20 °C for 60 days and then subjected to moist-sand germination on day 7 (3 replicates).

Fatty acid metabolomics analysis: the sample was thawed on ice and ground into powder. Then, 50 mg of the powder was placed in an Eppendorf tube, and 150 μL methanol, 200 μL methyl tert-butyl ether, and 50 μL of 36% phosphoric acid were added. The mixture was vortexed for 3 min, and then centrifuged at 4 °C and 12,000 rpm for 5 min. 200 μL of the supernatant was dried using a nitrogen evaporator (RapidVap N2, Labconco, Kansas, MO, USA). Then, 300 μL of a 15% boron trifluoride–methanol solution was added to the dry supernatant, and the mixtures was vortexed for 3 min and then dried in an oven at 60 °C for 30 min. After cooling to room temperature, 500 μL of an n-hexane solution and 200 μL of a saturated sodium chloride solution were added, vortexed for 3 min, and lastly centrifuged at 4 °C and 12,000 rpm for 5 min. We transferred 100 μL solution of the n-hexane layer to GC-MS analysis.

Sugar metabolism analysis: according to the method of Medeiros and Simoneit (2007) [82] and Sun et al. (2016) [83], the samples were freeze-dried under vacuum and then ground into powder using a ball mill (MM400, Retsch GmbH, Hahn, Germany) at 30 Hz for 1.5 min. Place 20 mg of the powder into a centrifuge tube and add 500 µL of the extraction solution (methanol: isopropanol: water (3:3:2, *v*/*v*/*v*)) to the tube. Vortex the mixture for three minutes, and then sonicate in an ice-water bath for 30 min. After this, the tube was centrifuged at 4 °C and 12,000 r/min for 3 min. Transfer 12.5 μL of the supernatant to another tube. Add 20 μL of an internal standard solution (250 μg/mL), then dry under a nitrogen stream (XD-DCY-24Y Shanghai Xida Instrument Co., Ltd. Shanghai, China), and freeze-dry in a freeze-dryer. Add 100 μL of methoxyammonium pyridine (15 mg/mL) to the tube and then incubate at 37 °C for 2 h. Subsequently, add 100 μL of BSTFA and incubate at 37 °C for 30 min to obtain the derivatized solution. Finally, add 800 μL of n-hexane to the derivatization solution, filter it through a 0.22 μm filter, and store the filtrate in a brown injection vial for GC-MS analysis.

Chromatographic and conditions for sugars and fatty acid analysis: Agilent 8890 gas chromatograph coupled to a 5977B mass spectrometer with a DB-5MS column (30 m length × 0.25 mm i.d. × 0.25 μm film thickness, J&W Scientific, Folsom, CA, USA) was employed for GC-MS analysis of sugars and fatty acid.

The oven-temperature ramp for sugars: 170 °C for 2 min, raised to 240 °C at a rate of 10 °C/min, raised to 280 °C at a rate of 5 °C/min, and then raised to 310 °C at a rate of 25 °C/min, 310 °C for 4 min. All other conditions are the same as for the determination of fatty acids. All samples were analyzed in selective ion monitoring mode. The ion source and transfer line temperature were 230 °C and 280 °C, respectively.

The oven-temperature ramp for fatty acid: 40 °C for 2 min, raised to 200 °C at a rate of 30 °C/min, 200 °C (1 min), raised to 240 °C at a rate of 10 °C/min, 240 °C for 1 min, and then raised to 285 °C at a rate of 5 °C/min, 285 °C for 3 min. Transfer line temperature: 240 °C. Ion source temperature: 230 °C. Quad temperature: 150 °C. Electron energy: 70 eV.

Orthogonal partial least squares discriminant analysis (OPLS-DA) was used for metabolites, with variable importance in projection (VIP) selection criteria (VIP > 1, *p* < 0.05, fold change (FC) ≥ 1.0 or ≤−1.0). Principal component analysis (PCA) was conducted using the prcomp R package (3.5.0). Using the Kyoto Encyclopedia of Genes and Genomes (KEGG) databases, we performed the pathway enrichment analysis.

KEGG compound databases were used to annotate identified metabolites (https://www.kegg.jp/kegg/compound/, accessed on 20 January 2025). Annotated metabolites were then mapped to the KEGG Pathway database (https://www.kegg.jp/kegg/pathway.html, accessed on 20 January 2025).

The pathways with significantly regulated metabolites were mapped and then fed into MSEA (metabolite sets enrichment analysis). We used the hypergeometric test’s *p*-values to determine their significances.

Sample preparation, extraction and analysis, metabolite identification, and quantification were performed by Wuhan Metaville Biotechnology Co., Ltd. (Wuhan, China).

### 4.4. Data Analysis

All experiments and measurements were performed in triplicate. Data were analyzed and figures were drawn using Prism 9.5 software and ggplot2 package of R software. Statistical analyses to assess significant differences between treatments were conducted using SPSS 23.0 (one-way ANOVA); *p* < 0.05, *p* < 0.01 and *p* < 0.001was considered statistically significant.

## 5. Conclusions

A high germination rate of 47.3% was observed after storing the seeds at −20 °C (LT) for 60 days, whereas the rate was only 32.7% at room temperature (25 °C) (RT). Direct GMS (germination in moist sand) was most effective among four germination methods.

The two stages of VI (after storage) and VII (the 7th day of germination) were crucial for releasing the dormancy and germination of *X. sorbifolium*. At stage VI, SEM revealed that the seed shells and kernels were of high integrity, particularly the kernels. Antioxidase activities, such as SOD, CAT, and POD, as well as the hormones such as IAA, IPA, and tHor/ABA, were found to be at higher levels. These metabolic levels ensured that the seeds reached an optimal state for dormancy release, making them ready for germination.

At stage VII, the optimal germination conditions (GMSs) created a suitable microenvironment, maintaining the higher levels of SOD, CAT, and POD, and kept ROS within signaling levels. Meanwhile, IAA, IPA, IAA/ABA, and tHor/ABA (increase 62.7%) maintained higher levels, and ABA decreased 23.2% in the LT treatment compared to the RT treatment. These environmental and metabolic levels facilitated the seed germination.

Low-temperature treatment promoted the degradation of starch and fats in the seeds. Significant accumulations of eight types of soluble sugars (e.g., glucose, D-fructose, allulose, and L-fucose) and three types of fatty acids (i.e., octanoic acid (C8-0), Cis-11,14,17-eicosatrienoic acid (C20-3n3), and γ-linolenic acid (C18-3n6)) were observed, while two types of soluble sugars and one type of fatty acid were downregulated under low-temperature conditions.

The results of this work indicated that low temperature as an external signal, together with ROS and GA-ABA-IAA etc. the internal signals co-regulated the seed dormancy release and germination of *X. sorbifolium*. Additionally, the moist-sand microenvironment was also a key factor in awakening the embryos of *X. sorbifolium* seeds.

## Figures and Tables

**Table 1:** Effects of different treatments on the seed germination and seedling quality of *X. sorbifolium*.

Storage Condition	Germination Treatment	Survival Rate/%	Height/cm	Main Root Length/cm	g (FW) /Seedling	g (DW) /Seedling
RT	GMS	39.8 ± 0.98 cd	7.8 ± 1.72 bcd	4.6 ± 0.86 bc	1.2 ± 0.34 a	0.26 ± 0.09 bcd
	35 °C + GMS	28.2 ± 2.18 e	6.0 ± 1.75 e	4.1 ± 1.48 c	1.0 ± 0.21 b	0.20 ± 0.04 d
	ABT + GMS	34.6 ± 4.49 d	7.1 ± 1.27 de	4.8 ± 1.07 b	1.1 ± 0.30 ab	0.23 ± 0.08 cd
	PEG + GMS	42.5 ± 3.18 bc	8.6 ± 1.70 bc	6.1 ± 1.40 a	1.2 ± 0.43 ab	0.28 ± 0.11 bcd
LT	GMS	60.7 ± 3.49 a	11.7 ± 2.13 a	7.1 ± 1.32 a	1.5 ± 0.31 b	0.34 ± 0.12 ab
	35 °C + GMS	36.5 ± 2.48 d	9.1 ± 2.70 bc	5.9 ± 0.86 ab	1.3 ± 0.55 ab	0.21 ± 0.05 cd
	ABT + GMS	45.9 ± 3.30 b	9.7 ± 1.91 b	6.2 ± 1.56 a	1.3 ± 0.35 ab	0.30 ± 0.07 bc
	PEG + GMS	65.7 ± 2.87 a	11.9 ± 3.07 a	6.8 ± 0.95 a	1.8 ± 0.45 a	0.39 ± 0.12 a

Data represent the mean ± SD of three biological replicates. Different lowercase letters indicate significant differences among same column at the *p* < 0.05 level. FW, fresh weight; DW, drought weight; GMS, direct germination in moist sand after storage; 35 °C + GMS, seed soaked in 35 °C water for 5 days after storage, then germinated in moist sand (the same below); ABT + GMS, seeds soaked in 200 mg/L ABT for 5 days after storage; PEG + GMS, seeds soaked in 5% PEG for 5 days after storage; RT, seeds stored at room temperature for 60 days; LT, seeds stored at −20 °C for 60 days.

**Table 2:** Differences in metabolites in sugars and fatty acid in *X. sorbifolium* seeds under two storage methods.

Compounds	Log_2_FC	*p*-Value	Type	Compounds	Log_2_FC	*p*-Value	Type
Trehalose	7.44	0.0015	up	Octanoic acid (C8-0)	1.56	0.0092	up
L-fucose	6.93	0.0004	up	Cis-11,14,17-eicosatrienoic acid (C20-3n3)	2.66	0.007	up
D-fructose	5.95	0.0002	up	Γ-linolenic acid(C18-3n6)	1.17	0.00019	up
Glucose	4.87	0.0003	up	Nonadecylic acid (C19-0)	−3.60	0.046	down
D-mannose	3.15	0.00004	up				
Maltose	2.88	0.0024	up				
D-xylose	2.48	0.000001	up				
2-Acetamido-2−deoxy-D-glucopyranose (ACE-D-G)	2.57	0.00003	up				
Raffinose	−1.42	0.0007	down				
D-galacturonic acid	−1.08	0.023	down				

## Data Availability

The original contributions presented in this study are included in the article/Supplementary Material. Further inquiries can be directed to the corresponding author.

## Supplementary Materials

The following supporting information can be downloaded at https://www.mdpi.com/article/10.3390/plants15182790/s1, Figure S1: The PCA of the metabolic profiles of seeds under two storage methods; Figure S2: The GC chromatograms for sugars and fatty acids; Table S1: The KEGG pathway of sugars in *X. sorbifolium* seeds; Table S2: The KEGG pathway of fatty acids in *X. sorbifolium* seeds; Table S3: The class and standard equation of sugars in *X. sorbifolium* seeds; Table S4: The class and standard equation of fatty acids in *X. sorbifolium* seeds.

## Abbreviations

The following abbreviations are used in this manuscript:

LT	Low temperature
RT	Room temperature
GMS	Germination in moist sand
tHor/ABA	(IAA + GA3 + ZR + iPA)/ABA
ROS	Reactive oxygen species
