# Supplementary material for: Low Temperature–ROS–Hormones Co-Regulation of Seed Dormancy Release and Germination in Xanthoceras sorbifolium
Source: Plants (Basel). 2026 Sep 11;15(18):2790. doi: 10.3390/plants15182790 (PMC13610468; doi:10.3390/plants15182790)
Supplement: Supplementary file 1 [file plants-15-02790-s001.zip › plants-4504191-supplementary.pdf]

Low Temperature–ROS–Hormonal Co-regulation of Seed Dormancy Release and Germination in *Xanthoceras sorbifolium*

Yifan Wang, Na An, Ying Chen*, Qinxia Wu, Zhao Yang, Hao Cai, Jingjing Di

Supplementary Figure

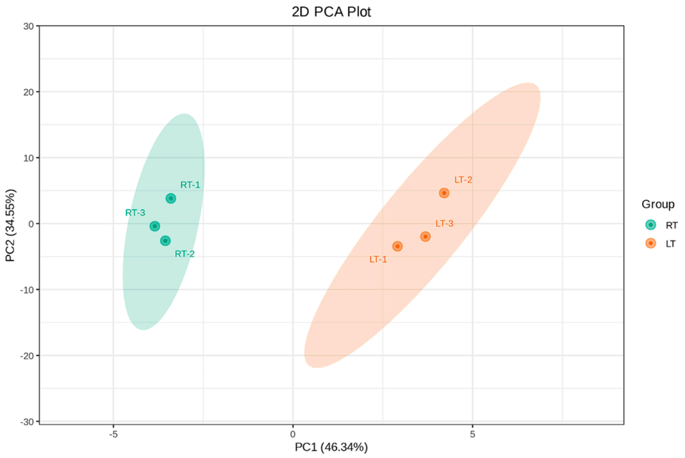

Figure S1. The PCA of the metabolic profiles of seeds under two storage methods

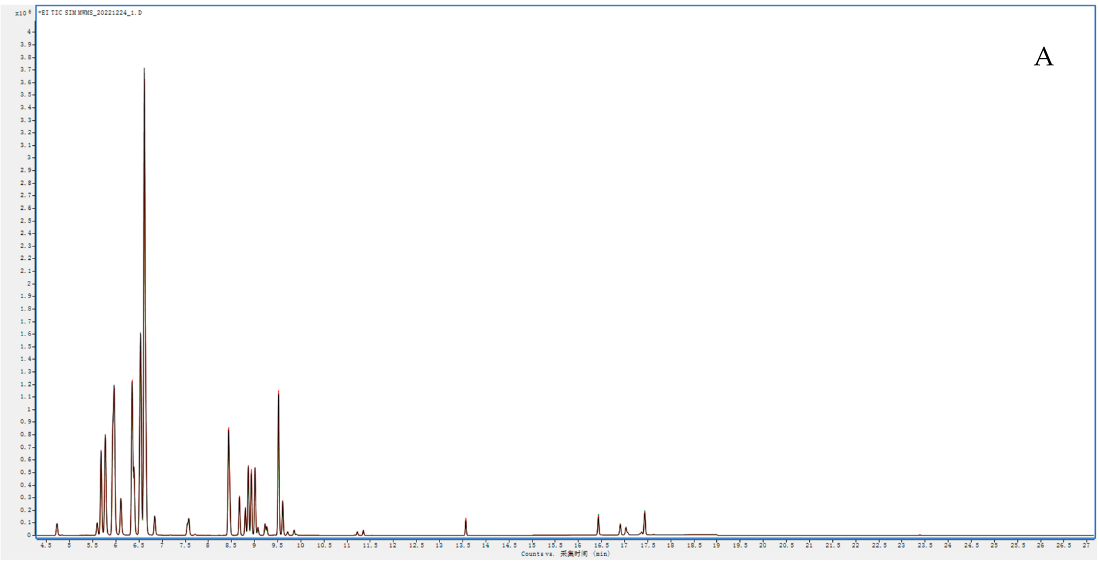

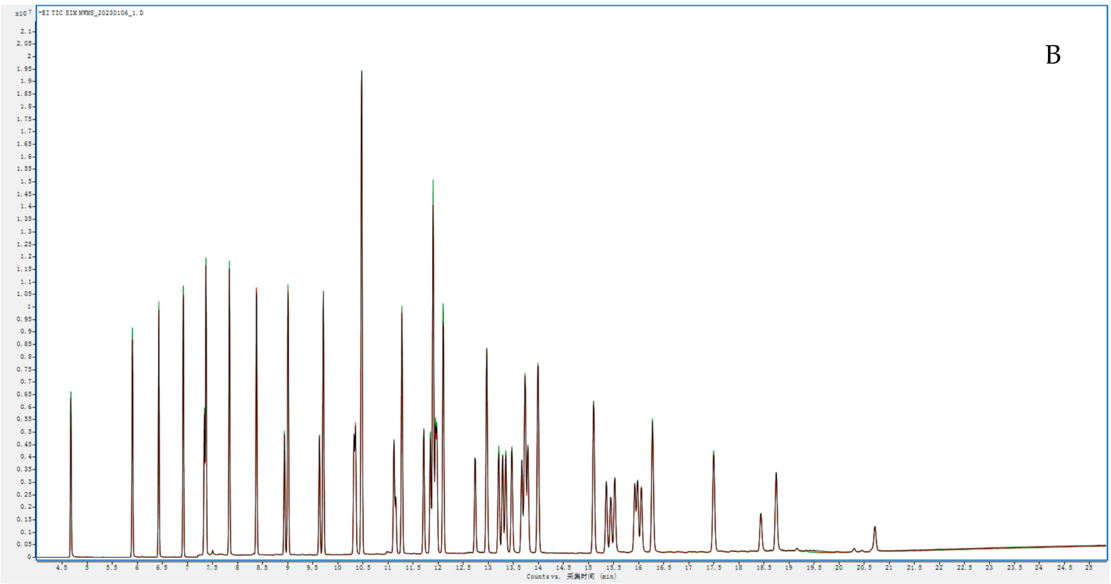

Figure S2. The GC- Chromatograms for sugars and fatty acids. A: sugars; B: fatty acids

Supplementary table

Table S1. The KEGG pathway of sugars in *X. sorbifolium* seeds

Kegg_pathway	ko_ID	Sig_ compound	Com- pound	Com- pound _all	Metabolites
Amino sugar and nucleotide sugar metabolism	ko00520	5	6	19	Fuc(up);2-Ace-2-Deo-D-Glucosamine(up);Xylose(up);Man(up);Glu(up)
ABC transporters	ko02010	8	14	19	Mal(up);Tre(up);2-Ace-2-Deo-D-Glucosamine(up);Xylose(up);Man(up);Glu(up);Gal-A(down);Raffinose(down)
Fructose and mannose metabolism	ko00051	3	5	19	Fuc(up);Man(up);Gal-A(down)
Biosynthesis of nucleotide sugars	ko01250	3	5	19	Fuc(up);2-Ace-2-Deo-D-Glucosamine(up);Man(up)
Starch and sucrose metabolism	ko00500	3	5	19	Mal(up);Tre(up);Glu(up)
Metabolic pathways	ko01100	9	18	19	Mal(up);Tre(up);Fuc(up);2-Ace-2-Deo-D-Glucosamine(up);Xylose(up);Man(up);Glu(up);Gal-A(down);Raffinose(down)
Biosynthesis of secondary metabolites	ko01110	3	5	19	Mal(up);Tre(up);Glu(up)
Pentose and glucuronate interconversions	ko00040	2	5	19	2-Ace-2-Deo-D-Glucosamine(up);Xylose(up)

Ascorbate and aldarate metabolism	ko00053	1	3	19	2-Ace-2-Deo-D-Glucosamine(up)
Biosynthesis of cofactors	ko01240	1	1	19	2-Ace-2-Deo-D-Glucosamine(up)
Galactose metabolism	ko00052	4	8	19	Man(up);Glu(up);Gal-A(down);Raffinose(down)
Glycolysis / Gluconeogenesis	ko00010	1	1	19	Glu(up)
Pentose phosphate pathway	ko00030	1	1	19	Glu(up)
Neomycin, kanamycin and gentamicin biosynthesis	ko00524	1	1	19	Glu(up)
Indole alkaloid biosynthesis	ko00901	1	1	19	Glu(up)

Table S2. The KEGG pathway of fatty acids in *X. sorbifolium* seeds

Kegg_pathway	ko_ID	Sig_ compound	Com- pound	Com- pound _all	Metabolites
Linoleic acid metabolism	ko00591	1	3	14	C18-3n6(up)
Biosynthesis of cofactors	ko01240	1	1	14	C8-0(up)
Lipoic acid metabolism	ko00785	1	1	14	C8-0(up)
Biosynthesis of unsaturated fatty acids	ko01040	1	9	14	C18-3n6(up)
Fatty acid biosynthesis	ko00061	1	7	14	C8-0(up)
Metabolic pathways	ko01100	2	10	14	C8-0(up);C18-3n6(up)
Biosynthesis of unsaturated fatty acids	ko01040	1	9	14	C18-3n6(up)

Table S3 The class and standard equation of sugars in *X. sorbifolium* seeds

**Index**	**Class**	**RT**	**Equation**	**R**	**Weighting**	**LLOQ**	**ULOQ**
2-Deo-ribose	monosaccharide	4.728	y = 0.067309 x + 1.192615E-005	0.992665613	1/x	0.001	0.2
Xylose	monosaccharide	5.684	y = 0.460980 x - 3.587170E-005	0.998892661	1/x	0.001	2

D-Ara	monosaccharide	5.775	y = 0.570273 x - 5.698041E-005	0.993946603	1/x	0.001	0.5
Xylulose	monosaccharide	5.959	y = 1.278523 x + 0.003792	0.99955921	1/x	0.002	4
Ribose	monosaccharide	5.975	y = 0.912350 x + 9.017135E-006	0.999564494	1/x	0.001	20
Ribono-1-4-lactone	monosaccharide	6.108	y = 0.432123 x + 2.248594E-004	0.995241048	1/x	0.001	2
Xylitol	monosaccharide	6.358	y = 0.524291 x - 1.696201E-005	0.997074224	1/x	0.001	1
Lev	monosaccharide	6.403	y = 0.139650 x + 4.404979E-005	0.998604215	1/x	0.001	0.2
Rha	monosaccharide	6.53	y = 0.131638 x + 2.793183E-006	0.996118912	1/x	0.001	1
Arabinitol	monosaccharide	6.547	y = 0.459943 x + 2.517916E-004	0.99827993	1/x	0.001	0.2
Fuc	monosaccharide	6.836	y = 0.721722 x + 9.760858E-005	0.999163445	1/x	0.001	2
Deo	monosaccharide	7.574	y = 0.100937 x - 6.209691E-006	0.995370501	1/x	0.001	1
1-5-Anh	monosaccharide	8.435	y = 0.635559 x - 3.464801E-005	0.99618339	1/x	0.001	0.5
Met	monosaccharide	8.438	y = 2.695741 x - 3.084577E-004	0.994049706	1/x	0.001	1
Fru	monosaccharide	8.672	y = 0.403247 x - 1.983430E-004	0.993489023	1/x	0.005	1
Man	monosaccharide	8.863	y = 0.528841 x - 2.571229E-005	0.996504214	1/x	0.001	0.2
Gal	monosaccharide	8.929	y = 0.458539 x - 2.704098E-005	0.996009076	1/x	0.001	0.2

Glu	monosaccharide	9.007	y = 0.668230 x - 0.001858	0.990910878	1/x	0.02	1
Glucuronic-A	monosaccharide	9.517	y = 0.359022 x - 1.871872E-005	0.992135923	1/x	0.001	0.5
Sorbitol	monosaccharide	9.52	y = 0.242741 x - 2.630661E-005	0.992740231	1/x	0.001	0.5
Gal-A	monosaccharide	9.606	y = 0.345698 x - 3.934911E-006	0.997404824	1/x	0.001	0.2
2-Ace-2-Deo-D-Glucosamine	monosaccharide	11.22	y = 0.056910 x - 1.801546E-007	0.998492222	1/x	0.001	2
Inositol	monosaccharide	11.35	y = 0.261693 x + 5.730210E-006	0.996911664	1/x	0.0002	1
Ribose-5-pho-Ba	monosaccharide	11.535	y = 0.014122 x - 2.205612E-005	0.997476081	1/x	0.02	1
Man-6-pho	monosaccharide	13.529	y = 0.041249 x - 4.964779E-005	0.998035516	1/x	0.01	1
Phe	disaccharide	13.565	y = 0.249414 x - 2.891439E-005	0.995156806	1/x	0.001	0.5
Suc	disaccharide	16.434	y = 0.190450 x - 0.028115	0.99224536	1/x	1	20
Lac	disaccharide	16.909	y = 0.063266 x + 6.518713E-006	0.99450905	1/x	0.001	0.2
Cel	disaccharide	17.032	y = 0.131870 x - 9.632782E-006	0.993576715	1/x	0.001	1
Mal	disaccharide	17.369	y = 0.097759 x - 6.990191E-006	0.997405075	1/x	0.001	2
Tre	disaccharide	17.436	y = 0.524140 x - 4.560868E-005	0.994503574	1/x	0.001	0.5
Raffinose	trisaccharide	23.388	y = 0.060274 x - 0.001640	0.992260464	1/x	0.2	5

Table S4 The class and standard equation of fatty acids in *X. sorbifolium* seeds

Index	Class	RT	Equation	R	Weighting	LLOQ	ULOQ
C6-0	lipid	4.667083333	y = 5883761.566605 x + 14342.120500	0.996873625	1/x	0.01	5
C8-0	lipid	5.90005	y = 77065.518179 x + 156.607610	0.997472503	1/x	0.01	5
C9-0	lipid	6.424633333	y = 146652.545780 x + 244.874762	0.997471304	1/x	0.01	5
C10-0	lipid	6.914766667	y = 230137.146122 x + 350.770325	0.997306517	1/x	0.01	5
C11-1 (cis-10)	lipid	7.336583333	y = 2013044.805808 x - 4346.105817	0.99916761	1/x	0.01	2
C11-0	lipid	7.364916667	y = 5986127.675387 x - 922.922563	0.99990948	1/x	0.01	2
C12-0	lipid	7.832416667	y = 460394.453749 x - 250.092105	0.998037291	1/x	0.01	5
C13-0	lipid	8.37785	y = 531696.662144 x + 1036.270877	0.991729784	1/x	0.01	10
C14-1	lipid	8.930366667	y = 139660.203892 x + 604.922278	0.994460352	1/x	0.01	10
C14-0	lipid	9.008283333	y = 662185.610277 x + 1394.701464	0.991746333	1/x	0.01	10
C15-1	lipid	9.63165	y = 140650.738974 x - 40.896564	0.995472249	1/x	0.01	10
C15-0	lipid	9.709566667	y = 790038.674384 x + 656.530194	0.994167667	1/x	0.01	10
C16-1	lipid	10.32583333	y = 145234.739117 x - 98.187453	0.991457245	1/x	0.01	10
C16-1T	lipid	10.35416667	y = 168417.534487 x - 861.105111	0.990774551	1/x	0.01	10
C16-0	lipid	10.47458333	y = 915358.190790 x + 3148.844471	0.995771373	1/x	0.02	10
C17-1	lipid	11.11918333	y = 132005.144602 x + 591.141835	0.991315352	1/x	0.01	20
C17-1T	lipid	11.1546	y = 70854.877287 x + 185.549895	0.996790368	1/x	0.01	20
C17-0	lipid	11.2821	y = 908861.751981 x + 3289.780085	0.991040078	1/x	0.01	20

C18-3n6	lipid	11.7142	y = 137605.284465 x + 90.492914	0.992697017	1/x	0.01	30
C18-2n6c	lipid	11.84878333	y = 318761.368627 x + 77.101680	0.995666659	1/x	0.01	30
C18-3n3	lipid	11.89838333	y = 153367.514827 x - 317.165927	0.997449926	1/x	0.01	5
C18-1n9c	lipid	11.90546667	y = 161448.293954 x + 423.242238	0.997399334	1/x	0.01	5
C18-2n6t	lipid	11.90546667	y = 454807.639317 x - 925.172904	0.996616344	1/x	0.01	5
C18-1n9t	lipid	11.95505	y = 142998.801427 x + 62.707332	0.996872288	1/x	0.01	30
C18-1(trans-11)	lipid	11.9763	y = 152714.562322 x - 405.143943	0.997399589	1/x	0.01	10
C18-0	lipid	12.1038	y = 1079945.685818 x + 1015.289859	0.996248232	1/x	0.01	10
C19-1(cis-10)	lipid	12.74131667	y = 113764.169648 x + 796.354680	0.996354051	1/x	0.01	20
C19-0	lipid	12.97506667	y = 1090268.280771 x - 367.599247	0.996406582	1/x	0.01	20
C16-2	lipid	13.20883333	y = 1030678.869439 x - 1077.764638	0.994784326	1/x	0.01	30
C20-4n6	lipid	13.29383333	y = 14562.439334 x - 42.828824	0.997398248	1/x	0.01	30
C20-5n3	lipid	13.35758333	y = 2943.312623 x + 174.912548	0.992985869	1/x	0.05	5
C20-3n6	lipid	13.478	y = 164079.725200 x - 959.456723	0.998952176	1/x	0.01	20
C20-2	lipid	13.67635	y = 306900.584051 x - 1838.144080	0.999252691	1/x	0.01	20
C20-1(cis-11)	lipid	13.73301667	y = 105890.230303 x + 34.491527	0.998189413	1/x	0.01	10
C20-3n3	lipid	13.74718333	y = 142478.965102 x - 245.604231	0.995450294	1/x	0.01	20
C20-1T	lipid	13.79676667	y = 126470.799849 x - 519.861888	0.99752437	1/x	0.01	10
C20-0	lipid	13.9951	y = 1131008.279118 x + 2340.482091	0.996539964	1/x	0.01	20

C21-0	lipid	15.10721667	y = 1155482.019904 x - 5977.005760	0.998733165	1/x	0.01	20
C22-6n3	lipid	15.35515	y = 2420590.433667 x - 48359.018994	0.998155484	1/x	0.02	30
C22-4(cis-7,10,13,16)	lipid	15.45431667	y = 21940.333265 x - 42.783095	0.997446323	1/x	0.01	30
C22-5(cis-7,10,13,16,19)	lipid	15.53223333	y = 4641.271778 x + 1883.980523	0.995306064	1/x	0.2	30
C22-2	lipid	15.92891667	y = 243515.432978 x - 1548.314916	0.996441716	1/x	0.01	30
C22-1n9	lipid	15.98558333	y = 77170.433060 x - 119.192160	0.999593083	1/x	0.01	30
C22-1T	lipid	16.05641667	y = 77244.778275 x - 469.797709	0.998731962	1/x	0.01	20
C22-0	lipid	16.28308333	y = 1121296.656717 x - 4333.529429	0.998716336	1/x	0.01	20
C23-0	lipid	17.50145	y = 982528.169391 x + 30331.203444	0.996261388	1/x	0.01	30
C24-1	lipid	18.44356667	y = 43840.341435 x - 396.196964	0.997578777	1/x	0.01	30
C24-0	lipid	18.74816667	y = 787836.849751 x - 7008.685016	0.995504115	1/x	0.01	30
